# Insight into planktonic protistan and fungal communities across the nutrient-depleted environment of the South Pacific Subtropical Gyre

**DOI:** 10.1128/spectrum.03016-23

**Published:** 2024-02-09

**Authors:** Katarina Kajan, Bernhard M. Fuchs, Sandi Orlić

**Affiliations:** 1 Division of Materials Chemistry, Ruđer Bošković Institute, Zagreb, Croatia; 2 Center of Excellence for Science and Technology-Integration of Mediterranean Region (STIM), Zagreb, Croatia; 3 Department of Molecular Ecology, Max Planck Institute for Marine Microbiology, Bremen, Germany; University of Minnesota Twin Cities, St. Paul, Minnesota, USA

**Keywords:** South Pacific Subtropical Gyre, parasitic protists, fungi

## Abstract

Ocean microorganisms constitute ~70% of the marine biomass, contribute to ~50% of the Earth’s primary production, and play a vital role in global biogeochemical cycles. The marine heterotrophic and mixotrophic protistan and fungal communities have often been overlooked mainly due to limitations in morphological species identification. Despite the accumulation of studies on biogeographic patterns observed in microbial communities, our understanding of the abundance and distribution patterns within the microbial community of the largest subtropical gyre, the South Pacific Gyre (SPG), remains incomplete. Here, we investigated the diversity and vertical composition of protistan and fungal communities in the water column of the ultra-oligotrophic SPG. Our results showed apparent differences in protistan community diversity in the photic and aphotic regions. The entire protistan community diversity was significantly affected by temperature, salinity, oxygen, and nutrient concentrations, while the parasitic community diversity was also affected by chlorophyll *a* concentration. The parasitic protists were assigned to the class Syndiniales accounting for over 98% of the total parasitic protists, exhibiting higher relative sequence abundance along the water depth and displaying consistent patterns among different sampling stations. In contrast to the protistan community, the fungal community along the SPG primarily clustered based on the sampling station and pelagic zones. In particular, our study reveals a significant presence of parasitic protists and functionally diverse fungi in SPG and their potential impact on carbon cycling in the gyre.

**IMPORTANCE:**

Our findings carry important implications for understanding the distribution patterns of the previously unrecognized occurrence of parasitic protists and functionally diverse fungi in the nutrient-limited South Pacific Gyre. In particular, our study reveals a significant presence of parasitic Syndiniales, predominantly abundant in the upper 300 m of the aphotic zone in the gyre, and a distinct presence of fungal communities in the aphotic zone at the central part of the gyre. These findings strongly suggest that these communities play a substantial role in yet insufficiently described microbial food web. Moreover, our research enhances our understanding of their contribution to the dynamics of the food webs in oligotrophic gyres and is valuable for projecting the ecological consequences of future climate warming.

## INTRODUCTION

In the marine environment, different microorganisms, including prokaryotes, protists, fungi, and viruses, have adapted to thrive in specific habitats, displaying a variety of forms, functions, and strategies (1, 2). These microorganisms constitute nearly 90% of the biomass and drive approximately 98% of primary production, playing a vital role in maintaining the structure and functioning of marine ecosystems (3–5). Studies have indicated that marine protists contribute to around 50% of annual primary production, consuming nearly 66% of it and an additional 10% of bacterial primary production (2, 6). Marine protists form a diverse and heterogeneous community consisting of autotrophs (primary producers), heterotrophs (phagotrophs and parasites), and a wide range of lineages that exhibit different mixotrophic strategies (7, 8). These different functional groups occupy specific niches within the marine food web and play crucial roles in biogeochemical cycles encompassing the majority of eukaryotic diversity in the oceans (2, 9). In contrast, the significance of marine ecosystems as fungal habitats has often been neglected (10–12). Recent advancements in methodologies have shed light on the role of fungi in various aquatic systems, but a comprehensive conceptual framework is still lacking (13, 14). However, it has been proposed that fungi have the capacity to greatly influence the structure, stability, and functionality of marine food webs through symbiotic and parasitic interactions with other organisms and degradation of organic matter (15–17).

One significant feature of the marine ecosystem is the presence of subtropical gyres, which cover approximately 70% of the ocean surface (18). Although the primary production per unit of the surface within these gyres is relatively low (19–21), their extensive size contributes significantly to the overall ocean productivity and global biogeochemical cycles (22). The microbial community’s activity, as revealed by both remote sensing and *in situ* measurements, plays a substantial role in shaping global biogeochemical cycles (23–26). Despite the numerous studies focusing on biogeographic patterns in marine ecosystems observed in microbial communities, our current understanding of the abundance and distribution patterns within the pelagic microbial community of the largest subtropical gyre, the South Pacific Gyre (SPG), remains limited. In contrast, numerous studies have been conducted in the water column of the open-ocean North Pacific Gyre, revealing the variety of microbial communities [i.e., references (27, 28)].

**Fig 1:**
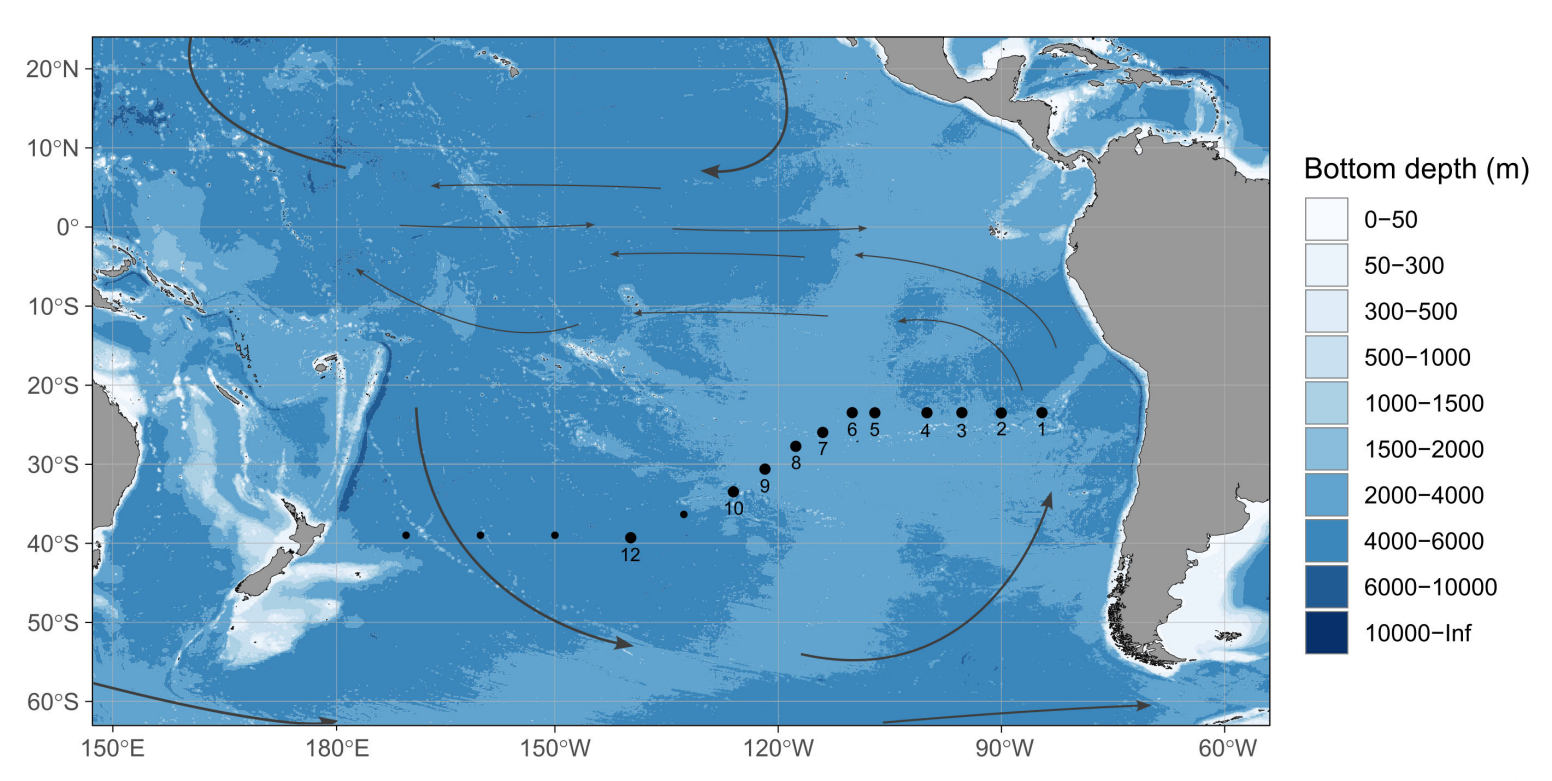
Map of sampling stations along the SO245 UltraPac transect. Black points denote sampling stations; samples used in the following study were collected at the enumerated dots. Gray arrows show the main ocean currents. The map was made in R and finished in Inkscape.

One of the sampling campaigns conducted in SPG was carried out at 15 stations along a transect from Antofagasta (Chile) to Wellington (New Zealand) during the R/V Sonne “UltraPac” cruise (SO245) in the Austral summer of 2015/2016 (Fig. 1). The most significant variations in physicochemical conditions were observed within the top 500 m of the SPG (Fig. S1) (29). In the central gyre region, surface water temperatures were consistently high, ranging between 20°C and 25°C (Fig. S1A). Chlorophyll fluorescence measurements indicated extremely low levels of primary productivity in the surface waters down to a depth of 70 m (Fig. S1B). The deep chlorophyll maximum (DCM) occurred between depths of 190 to 200 m, with a fluorescence reading of 0.5 µg L^−1^. Previous studies conducted in the same sampling campaign have analyzed prokaryotic and phototrophic eukaryotic communities together with dissolved organic matter (29–32). We aimed to decipher the diversity and vertical distribution of protistan and fungal communities in the water column of the ultra-oligotrophic SPG across the longitudinal scale of 5,500 km. In the central gyre region, the additional water sampling was carried out within the vertical profile of 300 m over 24 hours to evaluate the variation in the day-night cycle (diel shifts) of the protistan and fungal communities. Using a DNA metabarcoding approach (18S rRNA gene and ITS2 region), our objectives were to (i) uncover the longitudinal- and depth-related distribution patterns of protistan and fungal community in the water column, (ii) detect which physicochemical parameters shape the protistan and fungal community composition, and (iii) evaluate the dynamic diel variations in community structure along the water column in the central gyre region.

## RESULTS

### Community composition, diversity, and assembly of protists along the SPG

Based on the 18S rRNA gene amplicon sequencing data analysis, the protists found at different depths and stations along the SPG were clustered into 1,288 amplicon sequence variants (ASVs). The final data set included samples above 300 m encompassing the epipelagic, DCM, and upper mesopelagic zone, along with samples collected during diel sampling at station 8 (*n* = 84). Remarkably, along the SPG, the relative sequence abundance of the protistan community at the supergroup level exhibited a striking similarity of community structure across longitudinal scales of 5,500 km (Fig. S2). The level of protistan diversity in the epipelagic zone was higher than below the DCM in terms of ASV richness and Shannon index, showing a negative correlation to water depth (Table S1; Fig. S3). The highest (367 ASVs) and lowest (109 ASVs) richness values occurred at station 5 at 50 m and station 7 at 300 m depth, respectively (Table S1). On the contrary, no significant difference was found in the protistan phylogenetic distance along the water depth (Fig. S3).

**Fig 2:**
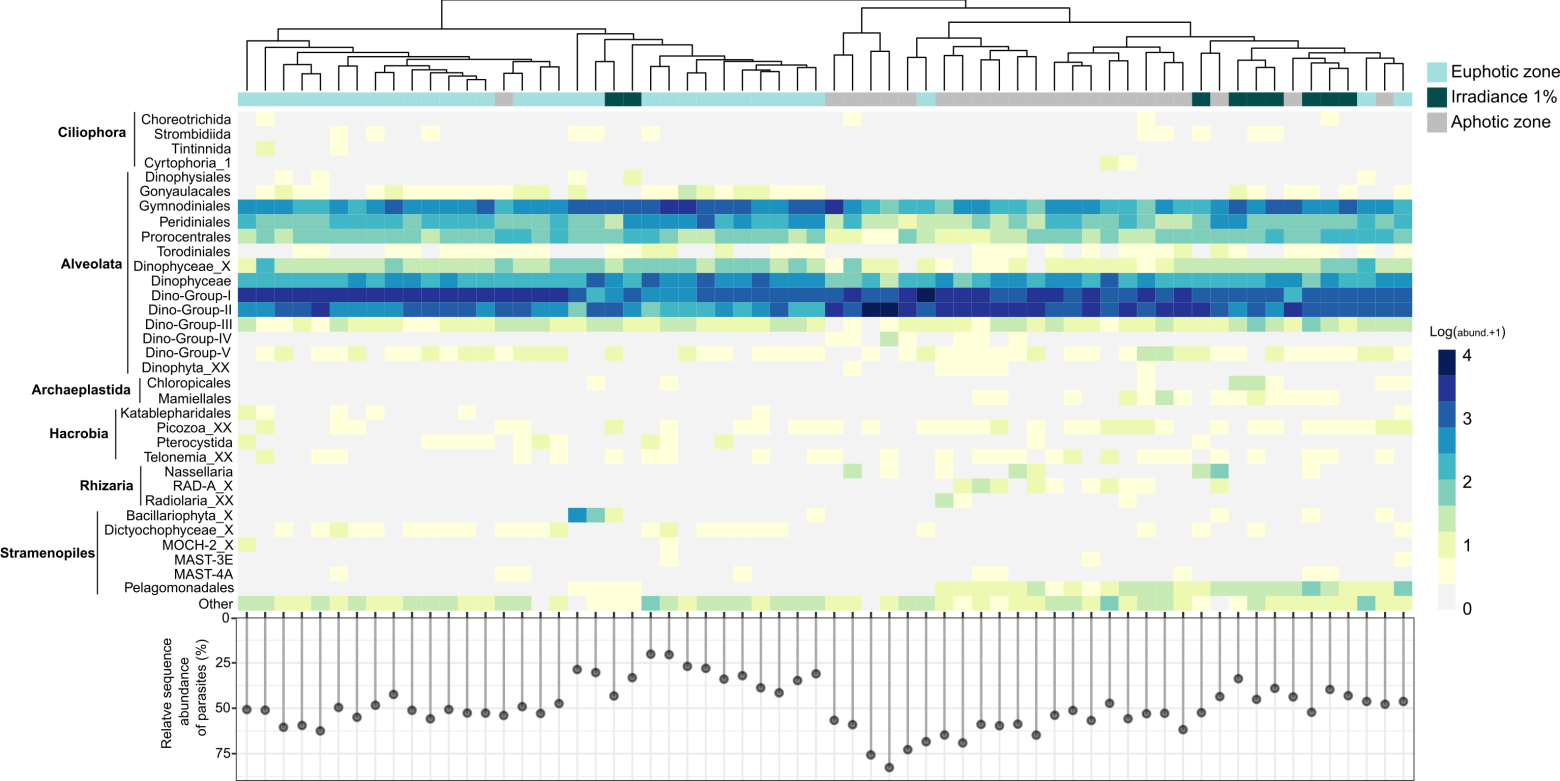
Partitioning of protistan major taxonomic groups by irradiance zone in South Pacific Gyre. Heatmap of relative sequence abundance of protistan community at the order level (orders with relative sequence abundance > 1%). Columns were clustered based on Bray-Curtis distance with the top row colored by irradiance zone. Abundances were log-transformed. Below the heatmap, the plot shows the relative sequence abundance of parasitic protists in clustered samples.

Alveolata dominated all analyzed samples, with an average relative sequence abundance of ~92% ± 2% within the protistan community per sampling station (Fig. S2). Other supergroups, such as Stramenopiles, did not excide a relative sequence abundance of 18%. Within the Alveolata, the division Dinoflagellates contributed significantly to protistan community composition (Fig. S4), together with 83% of the total assigned ASVs demonstrating relatively high ASV richness (total ASV *n* = 1,066; Table S1). These prevalent ASVs were taxonomically affiliated at the class level as Dinophyceae and parasitic Syndiniales, comprising 40% (25%–61%) and 48% (25%–68%) of the total ASV number, respectively (Fig. 2; Table S1; Fig. S5). Dinophyceae reads were particularly abundant in the euphotic zone, exhibiting a negative correlation with depth (Fig. S5). In contrast, the relative sequence abundance of Syndiniales increased in the aphotic zone (Fig. S5). These depth-related patterns of the protistan community composition were further supported by clustering the relative sequence abundance of the protistan community at the order level based on the different irradiance zones [Fig. 2; permutational multivariate analysis of variance (PERMANOVA), *R*^2^ = 0.280, *P* = 0.001].

**Fig 3:**
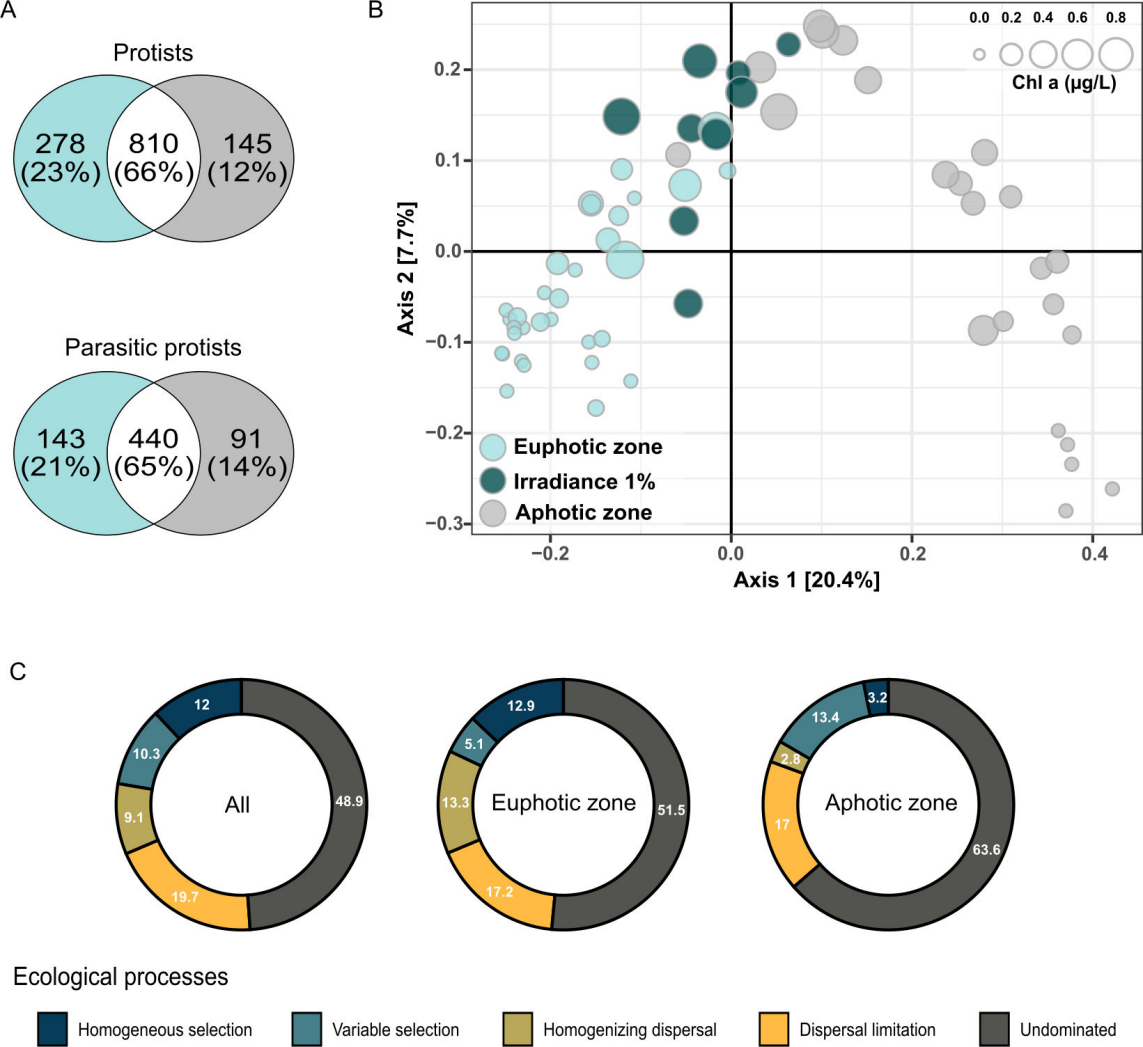
Vertical differences in the protistan community in South Pacific Gyre. (**A**) Number of unique, shared, and ubiquitous ASVs of all protists and parasitic protists across the photic and aphotic zones. Color-coded categories by irradiance zone (photic and aphotic zone). (**B**) Principal coordinate analysis (PCoA) of protistan community diversity color coded by irradiance zones, including irradiance zone of 1%. Each point represents an individual sample, and the circle size indicates the chlorophyll *a* concentration. (**C**) Relative importance of ecological processes driving the protistan assembly in SPG across the entire, euphotic, and aphotic zones.

On average, the protistan community accounted for 49% parasitic, 17% mixotrophic, 10% heterotrophic, and 5% photosynthetic protists in terms of functional groups (Fig. S6). Approximately, 18% of the community had unknown functions, primarily assigned only to the order level mainly to class Dinophyceae. Among the parasitic protists, 65% of the ASVs was found in both the euphotic and aphotic zones (Fig. 3A). However, a two-sample *t*-test revealed a significant difference in the relative sequence abundance of parasitic protists between the two zones with an average in the euphotic zone of 43.9% and in the aphotic zone of 58.5% (*t*-test, df = 51.6, *P* < 0.001; Table S1). The parasitic protists were represented by 667 ASVs assigned to the class Syndiniales, accounting for over 98% of the total parasitic protists. The remaining 2% of the ASVs was affiliated to parasitic protists Sagenista (Labyrinthulomycetes) and Ciliophora (Oligohymenophorea).

The parasitic Syndiniales encompassed five main groups at the order level: Dino-Groups I, II, III, IV, and V. The most abundant genera with high relative sequence abundance belonged to Dino-Groups I and II with 228 and 407 AVSs (Fig. 2; Table S1). Only the relative sequence abundance of Dino-Group II had a significant positive correlation with depth displaying consistent patterns among sampling stations (Fig. S7). In contrast, Dinophyceae had twofold less ASVs and a lower contribution of highly dominant genera to the overall protistan community (Table S1). At the order level, the most abundant with the highest relative sequence abundance were Gymnodiniales (15%), Peridiniales (7%), and Prorocentrales (5%) (Fig. 2).

The PCoA conducted at the ASV level demonstrated that the entire protistan community inhabiting the SPG primarily clustered based on sampling depth, exhibiting a distinct separation between the euphotic and aphotic zones (Fig. 3B). This result was further supported by PERMANOVA analysis, where sampling depth accounted for 42% of the variation of ASV composition, while the irradiance zone and sampling station contributed 19% each (Table S2). Notably, similar to the overall protistan community, the differences in sampling depth had a more pronounced impact on the structure of parasitic protists compared with zonal differences, confirming the significant influence of vertical physicochemical gradients on the overall composition of the protistan community.

The distance decay model was applied to identify the factors influencing the protistan diversity. The analysis revealed a significant similarity decline within the entire protistan and parasitic community as geographic distance increased (*R*^2^ = 0.019, *P* < 0.001; Fig. S8A; *R*^2^ = 0.036, *P* < 0.001; Table S3). Furthermore, linear regression models demonstrated that environmental differences had a stronger impact on the community structure diversity of the entire and parasitic protistan community compared with geographical distance (*R*^2^ = 0.313, *P* < 0.001; *R*^2^ = 0.194, *P* < 0.001; Table S3). The Mantel analysis was performed with various environmental factors to elucidate further the environmental drivers of protistan variation (Fig. S8B; Table S4). Results showed that the entire protistan community composition was significantly affected by temperature, salinity, oxygen, and nutrient concentrations, while the parasitic community additionally changed with chlorophyll *a* concentration (Table S4).

Whether the interplay of assembly processes governing the protistan community turnover varies in the euphotic and aphotic zone, the null model was applied (Fig. S9). The ecological processes across both zones of the SPG were dominated by dispersal limitation together with a higher influence of homogenizing dispersal and homogeneous selection in the euphotic zone (Fig. 3C). A great proportion of the processes was undominated (51%–63%).

### Diel variation of the protistan community composition

Evaluating the diel variation of the protistan community composition within the vertical profile of 300 m at the oligotrophic station in the central area of SPG (station 8), our results have shown that samples collected at the same depth at different time points have similar community structure while also showing the difference in irradiance zones (PERMANOVA; Fig. S10; Table S5). The significant inter-depth differences in protistan community structure were observed among communities at 50 m and 200 m and 100 m and 200 m, respectively [analysis of variance (ANOVA), *P* < 0.05; Fig. S11].

**Fig 4:**
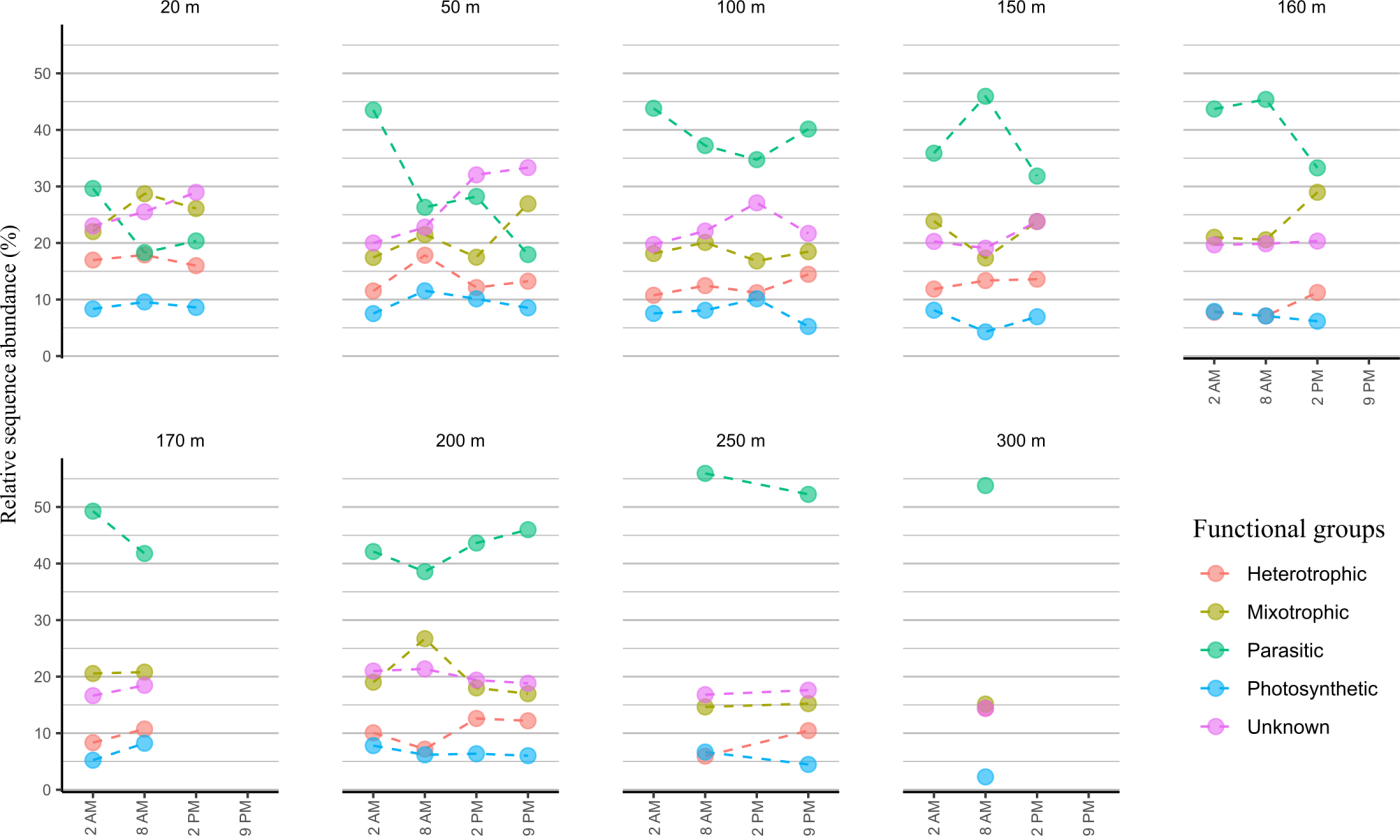
Diel variability in relative sequence abundance of protistan trophic functional groups at order level based on 18S rRNA gene amplicon sequencing data in the central gyre region at station 8 in the vertical profile of 300 m over 24 hours at four time points: 2 a.m., 8 a.m., 2 p.m., and 9 p.m.

The overall protistan community was predominantly composed of Alveolata (Dinoflagellata) across all samples (Fig. S11). A high negative correlation was shown between the relative sequence abundance of Syndiniales and Dinophyceae (Pearson’s correlation *R* = −0.97, *P* < 0.001). At station 8, Syndiniales were highly represented by Dino-Group I and Dino-Group II (Fig. S13). Within Dino-group I, clades 1 and 5 accounted for higher proportions than others, while clades 10 and 11 and 22 were the predominant clades in Dino-Group II. The relative sequence abundance of Dino-Group II exhibited a sharp increase in depth (Pearson’s correlation *R* = 0.88, *P* < 0.001). Dinophyceae were highly represented with Gymnodiniales and Peridiniales with the highest abundance at the surface (20 m; Fig. S13). The trophic functional groups exhibited contrasting distribution in diel depth variation (Fig. 4). The abundance of heterotrophic, mixotrophic, and photosynthetic protists reached the maximal relative sequence abundance in the euphotic zone, while parasitic communities in the aphotic zone (Fig. 4).

To distinguish diel patterns within the protistan community, the dynamic variations in protistan community structure in each sampled depth were evaluated based on the coefficient of variation (CV) within alpha diversity and relative sequence abundance at order and functional group levels (Fig. S14 to S16).

### Community composition and diversity of fungi along the SPG

Based on the ITS2 region, the relative sequence abundance of fungi was comparatively low to those of unidentified and unclassified, except for station 6 (Fig. S16). At station 6, the relative sequence abundance of fungi exceeded over 75% with vertical distribution across the mesopelagic and bathypelagic zones. For further analyses of the fungal community, a data set comprising 51 samples and 69 ASVs was used (constitutes 62% of the total ASVs assigned to fungi).

**Fig 5:**
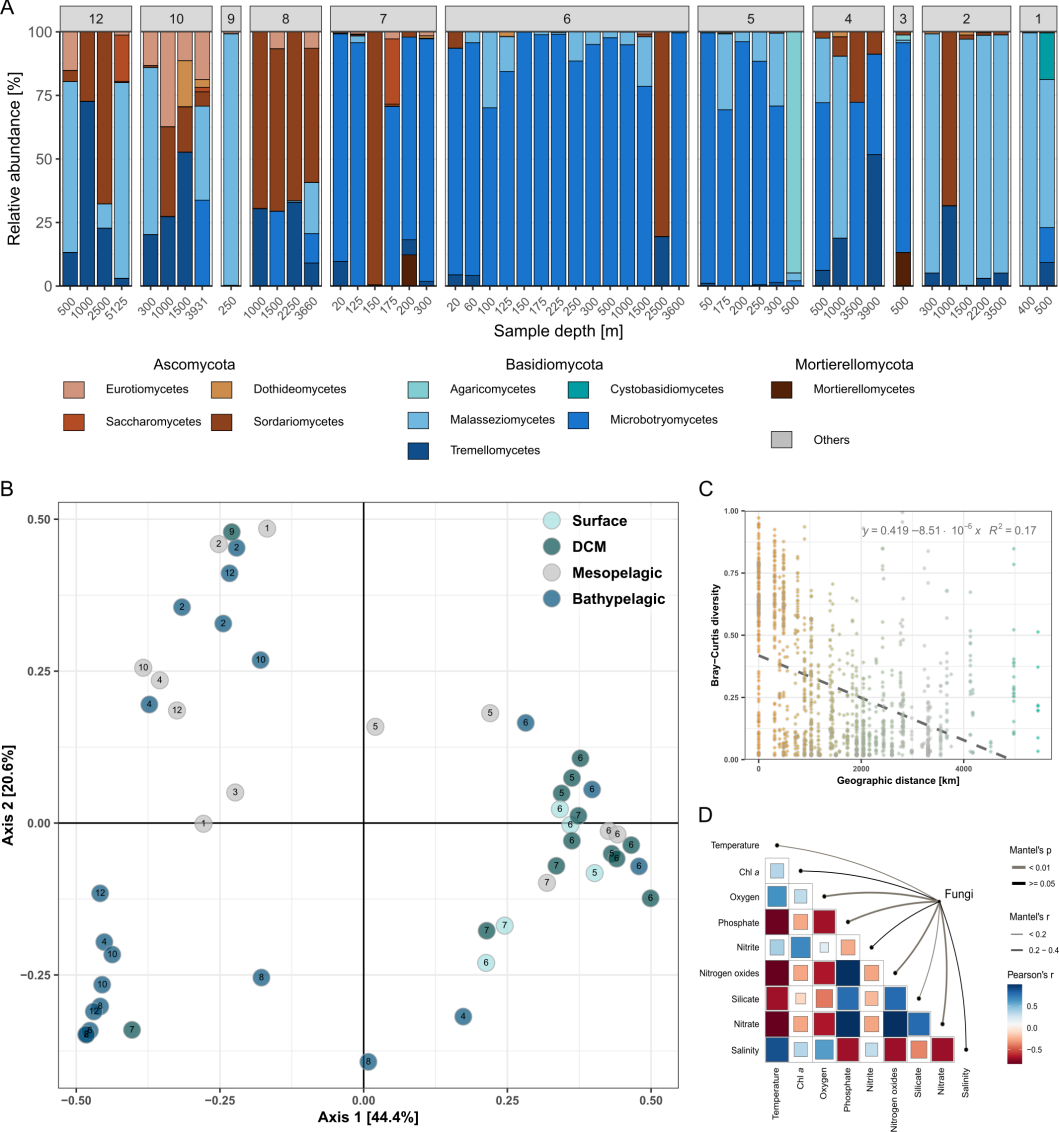
Partitioning of fungal major taxonomic groups and driving factors in South Pacific Gyre. (**A**) Relative sequence abundance of fungal classes per depth and sampling station. Classes with relative sequence abundance < 1% were aggregated into the group reported as “'Others.” (**B**) PCoA of fungal community diversity color coded by pelagic zones and number coded by the station. Pelagic zones: epipelagic (surface: 20–80 m, DCM: 100–250 m); mesopelagic: 300–500 m; and bathypelagic: 1,000–5,125 m. (**C**) Distance-decay relationship of Bray-Curtis and geographical distance (km) between sampling stations of fungal community at the ASV level (*P* < 0.001). The line represents a linear regression. (**D**) Mantel test correlation plot of fungi with environmental variables based on the Bray-Curtis distance.

The main phyla contributing to the fungal diversity of the SPG were Ascomycota, Basidiomycota, and Mortierellomycota, with 20, 47, and 2 ASVs, respectively (Fig. 5A). ASVs assigned to Basidiomycota exhibited high relative sequence abundances in most samples, while Ascomycota had higher relative sequence abundance at sampling stations 7, 8, 10 and 12. In contrast to the majority of samples, the fungal community in the central part of SPG (station 6) was present in all depths dominated by the genus *Rhodotorula* (Microbotryomycetes). A considerable fraction of the phylotypes assigned using FUNGuild were classified as phototrophs, while 37% of ASVs represented saprotrophs and symbiotrophs (Fig. S17).

In contrast to the protistan community, the PCoA analysis showed that the fungal community along the SPG primarily clustered based on the sampling station and pelagic zones (Fig. 5B; Table S6). The site-specific diversity was confirmed with a higher distance decay relationship of fungal diversity and geographical distance in comparison to the protistan community (*R*^2^ = 0.166, *P* < 0.001; Fig. 5C; Table S7). Conversely, the linear regression model demonstrated low environmental differences contributing to fungal diversity (*R*^2^ = 0.018, *P* < 0.001; Table S7). Nevertheless, parameters such as oxygen, nitrates, phosphates, and temperature, unlike salinity and chlorophyll *a*, strongly affected the fungal diversity (Fig. 5D; Table S8).

Further analysis of fungal diversity and environmental parameters at station 6 revealed no significant correlations. Due to the relatively low relative sequence abundance of fungal ASVs, the diel variation of the community based on the ITS2 region was not further analyzed.

## DISCUSSION

This study provides new insights into the vertical and longitudinal distribution of taxonomic and functional composition of protists and fungi along the subtropical South Pacific Gyre.

Taking place during the Australian summer, the research cruise covered a vast geographical area of 7,000 km along the SPG, conducting sampling that spanned the entire vertical profile from the surface to the depths of the bathypelagic waters. Our study comprised samples within the longitudinal scale of 5,500 km in which we analyzed the diversity and composition of the protistan and fungal community using 18S and ITS regions, respectively.

The similarity analysis revealed significant differences in the oceanic protist community regarding water depth. Depth was identified as a major driver of protist community composition, which aligns with previous studies (28, 33–36). The distribution pattern of protists closely resembled that of the bacterial community diversity studied in the same period within the SPG (29). Notably, the composition of the bacterial community exhibited substantial changes with increasing depth, primarily driven by variation in light variability (29). The most pronounced shifts in bacterial composition occurred in the 1% irradiance zone, characterized by a decline in the abundance of mesopelagic clades (29). A prior study on photosynthetic microbial communities found that the photosynthetic community primarily comprised *Prochlorococcus*, aerobic anoxygenic phototrophs, and a diverse community of small photosynthetic eukaryotes. Surprisingly, photosynthetic activity spanned the euphotic zone rather than concentrated solely at the DCM, with 68%–79% of primary productivity occurring above DCM (32) aligning with our observation.

In this study, we found significant correlations between environmental variables and protistan diversity, highlighting the impact of abiotic factors on community dynamics within the upper 300 m of the water column. Our results indicate that protistan diversity is primarily influenced by temperature and nitrogen availability rather than other environmental factors or geography. These results are consistent with the vertical stratification of microbial communities, which respond to changes in physicochemical parameters, such as light, temperature, and nutrient availability (37). Notably, the extremely low concentrations of inorganic nitrogen throughout the euphotic zone indicate the importance of nutrient recycling for primary production in the SPG. This emphasizes the crucial role of heterotrophic activities responsible for the breakdown and recycling of organic matter (32).

Dinoflagellates exhibit diverse metabolic capabilities, including photosynthetic, mixotrophic, and heterotrophic (parasitic). This versatility allows dinoflagellates to contribute to the export of organic matter through their photosynthetic activities in the euphotic zone and the remineralization processes within the mesopelagic zone within the same regional environment (38).

The aphotic zone of the SPG exhibited a greater prevalence of parasitic communities, indicating their heightened contribution to the overall ecosystem. However, despite the differences in relative sequence abundances, there was also a notable similarity between the communities. This similarity suggests that ecologically and morphologically similar species have likely partitioned their ecological niches, allowing for their coexistence in the region. A high prevalence of parasitic protists was also recorded in our diel sampling, with the highest contribution of Syndiniales. Their wide distribution and genetic diversity due to their ability to colonize a wide range of ecological niches, which was also represented in our results (39–43). Parasitism is an important source of mortality within marine protist communities, though it is seldom accounted for in ecosystem and biogeochemical models (44).

Additionally, previous research has shown that the mesopelagic zone exhibits high metabolic activity among protists and approximately 90% of carbon is respired in this particular layer. Syndiniales, highly abundant in our samples, appear to have a significant role as parasites in SPG food webs, although this interaction has been overlooked. This suggests a potential feedback mechanism between parasitic infections, the release of organic matter, and prokaryotic assimilative activity. The capacity of these parasitoids to control their hosts depends on their parasitic fitness and mechanisms that determine parasitic specificity (45, 46). Host density is thought to be the main determinant of parasite abundance and infection rates, with increased host encounters and infections occurring under conditions of a plankton bloom. Other factors may influence Syndiniales population dynamics, such as temperature, nutrients, water column depth, and degree of physical mixing, although these factors have not been studied extensively. In addition, zoosporic parasites likely play a significant role in maintaining genetic polymorphism and biodiversity in host populations and regulating phytoplankton succession. They can infect several types of plankton including protozoan, such as dinoflagellates or ciliates, and metazoans, such as crustaceans and copepods as endoparasites (47). Because of their short generation times and abundant progeny, zoosporic parasitoids exert important top-down control that significantly influences the entire aquatic food webs, especially phytoplankton population dynamics. Our limited diel resolution makes it difficult to identify reliable factors influencing Syndiniales populations under different ecological and biological conditions (48).

The global planktonic marine fungal community has been found to cluster by the ocean, suggesting that fungal dispersal occurs in oceans (49, 50). Fewer studies have focused on planktonic fungi in the open ocean compared with coastal regions, and even fewer have targeted the mesopelagic zone and below. Our results have shown the clustering of fungal communities among the sites and confirmed that site-to-site variation was a stronger factor in explaining fungal community structure, suggesting that local environmental filtering may play a critical role in assembling the fungal community in SPG (50). The homogeneous fungal community patterns observed in ultra-oligotrophic sampling station 6, the “oligotrophic eye,” reflect surprisingly stable community composition across the vertical depth profile from 20 to 3,600 m. The typically oligotrophic conditions would suggest low abundance of pelagic fungi, which may increase under eutrophication or dissolved organic carbon enrichment, as observed for copiotrophic bacteria. Although few studies have tried to quantify the actual biomass of fungi, it seems it could exceed that of bacteria, particularly in habitats rich in organic carbon (51–53). Additionally, the fungal community in the SPG inhabiting the aphotic realm was found to be closely related to especially oxygen and nitrate concentrations. The prevalent genus *Rhodotorula* detected in the SPG is the predominant basidiomycetes usually found in the marine environments such as seawater, sediment, and hydrothermal vent, and also as symbionts of marine invertebrates and seaweeds (51, 54, 55). New studies of deep-sea sediment fungi isolates in SPG have shown their metabolic capability of degradation of aromatic compounds, lignin, lignocellulose, carbonate, and carboxylic acids (56).

### Conclusions

Our study provides evidence of the previously unrecognized occurrence of parasitic protists and functionally diverse fungi in the nutrient-limited South Pacific Gyre. In this work, we have analyzed the protistan community focusing on the significant presence of parasitic Syndiniales, which were found to be particularly abundant in the upper 300 m of the aphotic zone in the SPG. Additionally, our analysis of fungal communities revealed their distinct presence in the aphotic zone at the central part of the SPG, suggesting their substantial contribution to the yet insufficiently described microbial food web. To draw broader conclusions, additional samplings are required to ensure significant reliability and enhance the applicability of our findings. Exploring the diversity of fungi and their interactions with protistan and prokaryotic communities will enable us to understand open ocean ecosystems comprehensively.

## MATERIALS AND METHODS

### Field sampling

A transect along the South Pacific Ocean covered a length of 7,000 km and a depth of up to 5,000 m. Sampling was carried out at 15 stations along a transect from Antofagasta (Chile) to Wellington (New Zealand) during the R/V Sonne “UltraPac” cruise (SO245) in the Austral summer from 17 December 2015 to 28 January 2016 (Fig. 1). The detailed sampling strategy, physicochemical measurements, and nucleic acid extraction methods are described by Reintjes et al. (29). Physical oceanography, oxygen, and nutrient data are available via the Pangea database (57, 58). The samples used in this study are listed and described in Table S9. Briefly, a total of 152 seawater samples were collected from multiple depths at 11 stations, including five intermediate stations: 1, 3, 5, 7, and 9, and six main stations: 2, 4, 6, 8, 10, and 12. Six sampling stations covered the central gyre region (stations 4–9; SPG), three sampling stations on the northern east side of the gyre (Southeast Pacific), and two stations in the Southwest Pacific. In total, 8 samples were collected throughout the water column on the intermediate stations in the range of 20 to 500 m, while 12 to 15 samples were collected on the main stations with a maximum depth of 50 to 100 m above the seafloor. Water sampling encompassed epipelagic (surface: 20–80 m, DCM: 100–250 m), mesopelagic (300–500 m), and bathypelagic zones (1,000–5,125 m). The depth of the irradiance zones (photic, irradiance of 1%, and aphotic zone) varied between the sampling stations. The depth of the zone with irradiance of 1% varied between 162 m in the SPG (stations 4 to 9), 110 m in station 1, and 94 m in station 12.

In the central gyre region at station 8, the additional water sampling was carried out within the vertical profile of 300 m over 24 hours to evaluate the variation in the day-night cycle (diel shifts) of the protistan and fungal communities. Water samples at nine depths were collected at four time points: 2 a.m., 8 a.m., 2 p.m., and 9 p.m.

### Amplicon sequencing and analysis

The V4 region of the 18S rRNA gene was amplified with primers TAReuk454FWD1 and TAReukREV3 (59) and the ITS2 region of the fungal rRNA gene with primer pair ITS3-Mix1-Mix2 (TCCTCCGCTTAyTgATAtGc), a modified ITS3 Mix2 forward primer ITS3-mkmix2 (CAWCGATGAAGAACGCAG) (60), and a reverse primer ITS4 [equimolar mix of cwmix1 (TCCTCCGCTTAyTgATAtGc) and cwmix2 (TCCTCCGCTTAtTrATAtGc)] (61), using a unique dual-barcoding two-step PCR approach (UDB-H12) as described by Pjevac et al. (62). Amplicons were sequenced in a paired-end mode (2 × 300 bp) on a MiSeq platform (Illumina, San Diego, CA, USA) at the Joint Microbiome Facility of the Medical University of Vienna and the University of Vienna. Details on sequence trimming and settings for quality filtering are described by Pjevac et al. (62). Sequence data were processed in R (63) using DADA2 following the workflow by Callahan et al. (64) in a pooled mode using all amplicon libraries per sequencing run. Taxonomic assignment was done by mapping 18S V4 ASV sequences against the PR2 reference database (v.4.12.0) (65) and ITS2 ASV sequences against the UNITE reference database (v. 04.02.2020) (66). The ASVs assigned to the protistan taxon at the taxonomic level “Division” were extracted from the 18S V4 data set, and the fungal taxon at the taxonomic level “Kingdom” extracted from the ITS2 data set was kept for further statistical analysis and data visualization. The functional groups were assigned based on the taxonomical classification of protistan ASVs into autotrophs, mixotrophs, parasites, osmotrophs, and phagotrophs. The functional assignment was determined according to Adl et al. (67) and the literature review (68, 69). The identified fungal taxa were assigned to functional groups with the FUNGuild tool (70) into pathotrophs, saprotrophs, symbiotrophs, etc. ASVs that did not match any taxa in the database were categorized as “Unknown.”

### Statistical analysis and data visualization

All analyses and visualizations were performed with R v 4.2.2 (63) in R studio using packages phyloseq (71), tidyverse (72), vegan (73), ggplot2 (74), pheatmap (75), ggVennDiagram (76), picante (77), linkET (78), enmSdm (79), and geosphere (80). Prior to analyses, the standardization method of the ASV tables was applied according to Gutiérrez-Rodríguez et al. (81). Samples collected during diel sampling at station 8 with four time points (2 a.m., 8 a.m., 2 p.m., and 9 p.m.) were analyzed separately. Specifically, only samples obtained at 2 p.m. at station 8 were included for comparison with those from other stations.

The alpha diversity was estimated as the richness, Shannon-Wiener index, and Faith’s phylogenetic distance computed using packages vegan and picante (73, 77). The phylogenetic tree of the protistan community was generated using Clustal Omega (82) for alignment and FastTree (83) to estimate phylogeny. Pearson’s correlation was used to measure the strength of the linear relationship between relative sequence abundance and each predictor variable. PCoA was performed on the Bray-Curtis distance matrix at the level of protistan and fungal ASV. Prior to beta diversity analysis, Hellinger transformation was applied to the community composition data sets. To test for the compositional differences of protistan and fungal communities between the sampling station, depth, and irradiance zone among the samples, a PERMANOVA was run with 999 permutations using the function *adonis2* in package vegan (73). Using linear regression, the distance-decay relationship was evaluated by comparing the Bray-Curtis community distance matrix of protistan and fungal communities with geographic (km) and environmental distance. The geographic distance matrix was measured between the geographical location of sampling stations [package enmSdm (79) and geosphere (80)]. The Mantel test was used to determine the driving environmental factors (Euclidean distance) on protistan and fungal community diversity based on the Bray-Curtis community distance matrix [linkET package (78)].

To evaluate the dynamic variation in community composition in each depth in the central gyre region at station 8, the CV was calculated. The average diel beta diversity based on the Bray-Curtis distance of community composition was calculated to obtain information on the inter- and intra-depth variabilities at four time points. The depth differences were tested by one-way ANOVA, followed by Tukey’s multiple comparison tests.

### Quantifying influences of ecological processes

The biodiversity ecological null model was used to evaluate processes driving protistan assembly (84, 85). Based on the standardized abundance ASV table and amplicon phylogenetic tree, the β-nearest taxon index (βNTI) of the protistan community was calculated. In order to test if there was a significant difference between molecular and phylogenetic turnover between the observed protistan assemblages, the β-mean nearest taxon index (βMNTD) was calculated. Further, the βNTI was calculated as the difference between the observed βMNTD and the null distribution. Deterministic processes (variable or homogeneous selection) are dominated when βNTI is greater than 2 or less than −2. Values within the 2 > NTI > −2 range indicate the dominance of stochastic processes (homogenizing dispersal or dispersal limitation) or random drift. Based on the sequence abundance of the protistan community, the Raup-Crick (RC) beta diversity was calculated to distinguish stochastic processes. Assemblies were structured by dispersal limitation if RC > +0.95, homogenizing dispersal if RC < −0.95, or random processes acting alone to undominated if RC falls between −0.95 and +0.95.

## Data Availability

The raw reads of the 18S rRNA gene and ITS2 region amplicon sequencing were deposited to the NCBI Sequence Read Archive (SRA) database under the BioProject accession number PRJNA1051843.

## SUPPLEMENTAL MATERIAL

Supplemental figures spectrum.03016-23-s0001.docx Figures S1 to S18.

Supplemental tables spectrum.03016-23-s0002.xlsx Tables S1 to S9.

ASM does not own the copyrights to Supplemental Material that may be linked to, or accessed through, an article. The authors have granted ASM a non-exclusive, world-wide license to publish the Supplemental Material files. Please contact the corresponding author directly for reuse.
