# Supplementary material for: Insight into planktonic protistan and fungal communities across the nutrient-depleted environment of the South Pacific Subtropical Gyre
Source: Microbiol Spectr. 2024 Feb 9;12(3):e03016-23. doi: 10.1128/spectrum.03016-23 (PMC10913754; doi:10.1128/spectrum.03016-23)
Supplement: Supplemental figures — Figures S1 to S18. [file spectrum.03016-23-s0001.docx]

Insight into planktonic protistan and fungal communities across the nutrient-depleted environment of the South Pacific Subtropical Gyre

Katarina Kajan^a,b^, Bernhard M. Fuchs^c^, Sandi Orlić^a,b,#^

^a^ Division of Materials Chemistry, Ruđer Bošković Institute, Zagreb, Croatia

^b^ Center of Excellence for Science and Technology-Integration of Mediterranean Region (STIM), Zagreb, Croatia

^c^ Department of Molecular Ecology, Max Planck Institute for Marine Microbiology, Bremen, Germany

Running Title: Protists and fungi in the South Pacific Gyre

# Address correspondence to Sandi Orlić [sorlic@irb.hr](mailto:sorlic@irb.hr)

**This file includes Supplementary figures** (Figures S1 to S18)


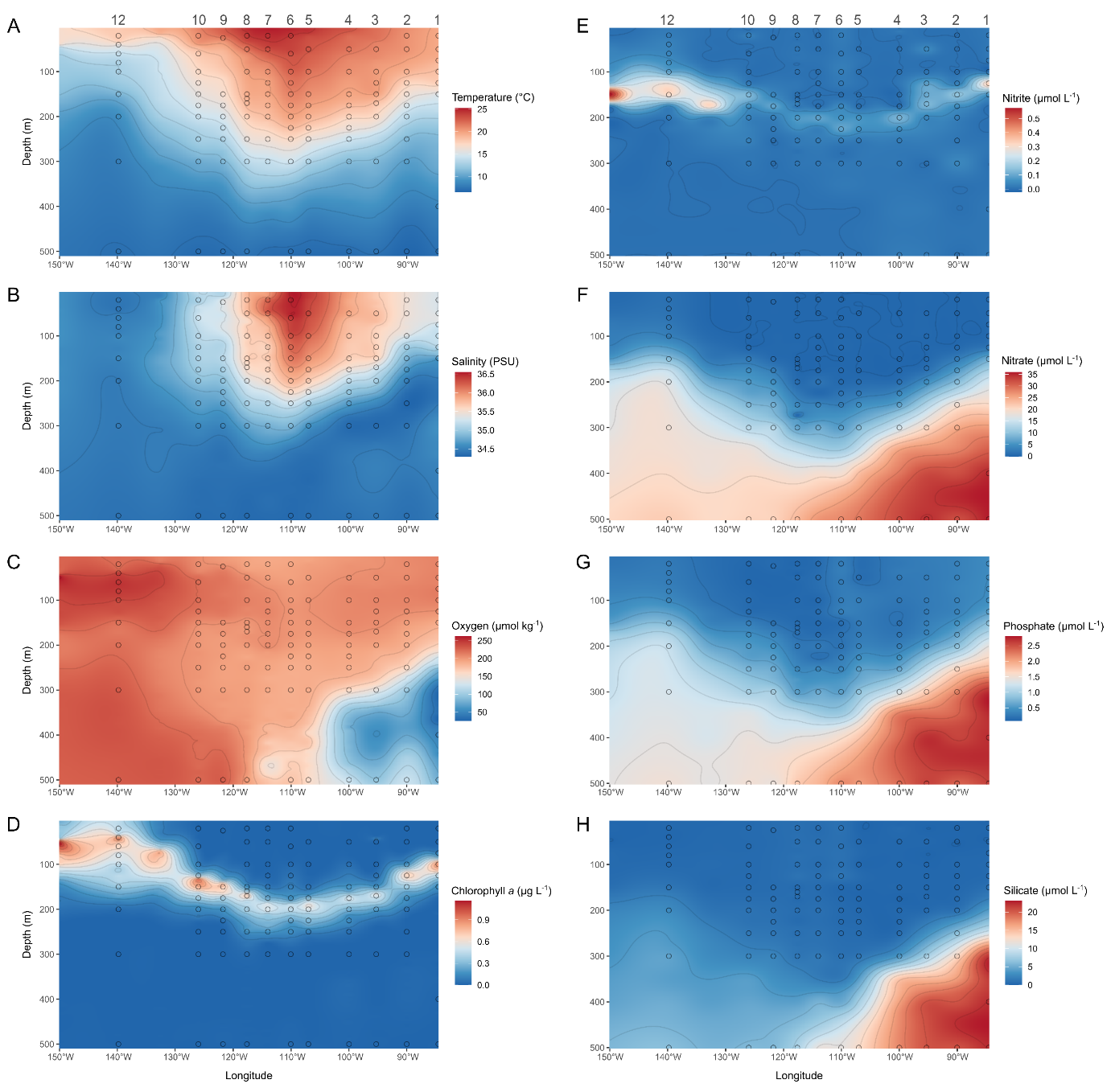


**Fig. S1** Distribution of physical and chemical parameters along the SO245 UltraPac transect. Transect distribution of: (A) temperature, (B) salinity, (C) oxygen, (D) chlorophyll *a*, (E) nitrite, (F) nitrate, (G) phosphate and (H) silicate. Circles mark sampling and measurement depths.


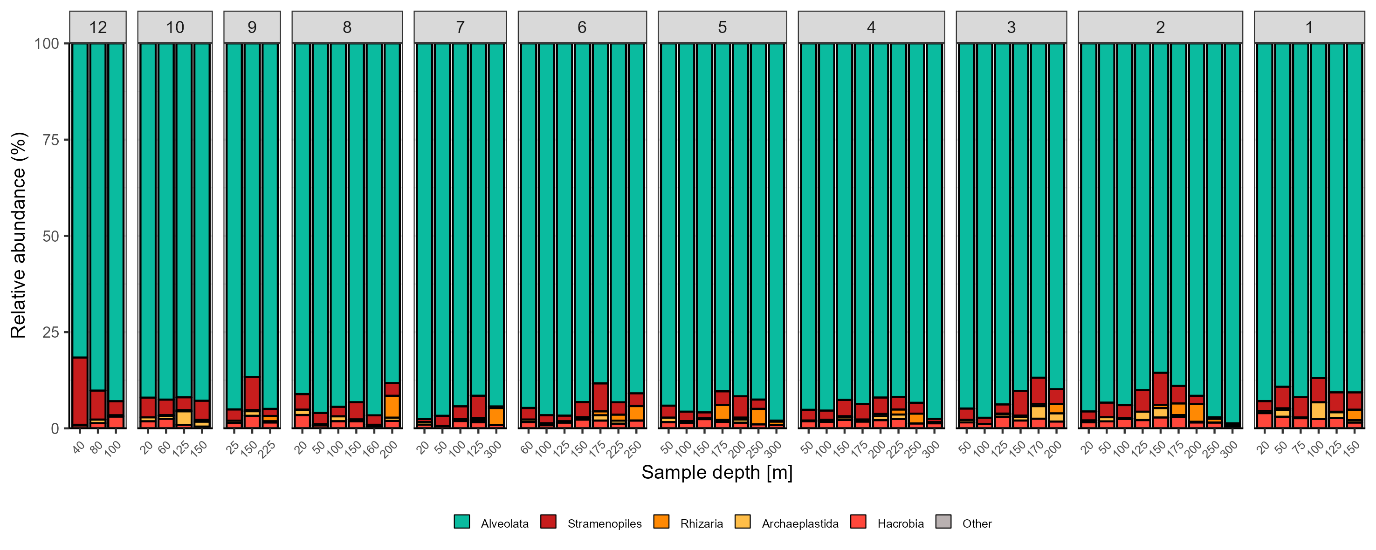


**Fig. S2** Depth distribution of protistan community composition per sampling station at supergroup level. The relative sequence abundance of supergroups based on 18S rRNA gene amplicon sequencing data within 20 to 300 m. Supergroups with relative sequence abundance <1% were aggregated into the group reported as "Other".


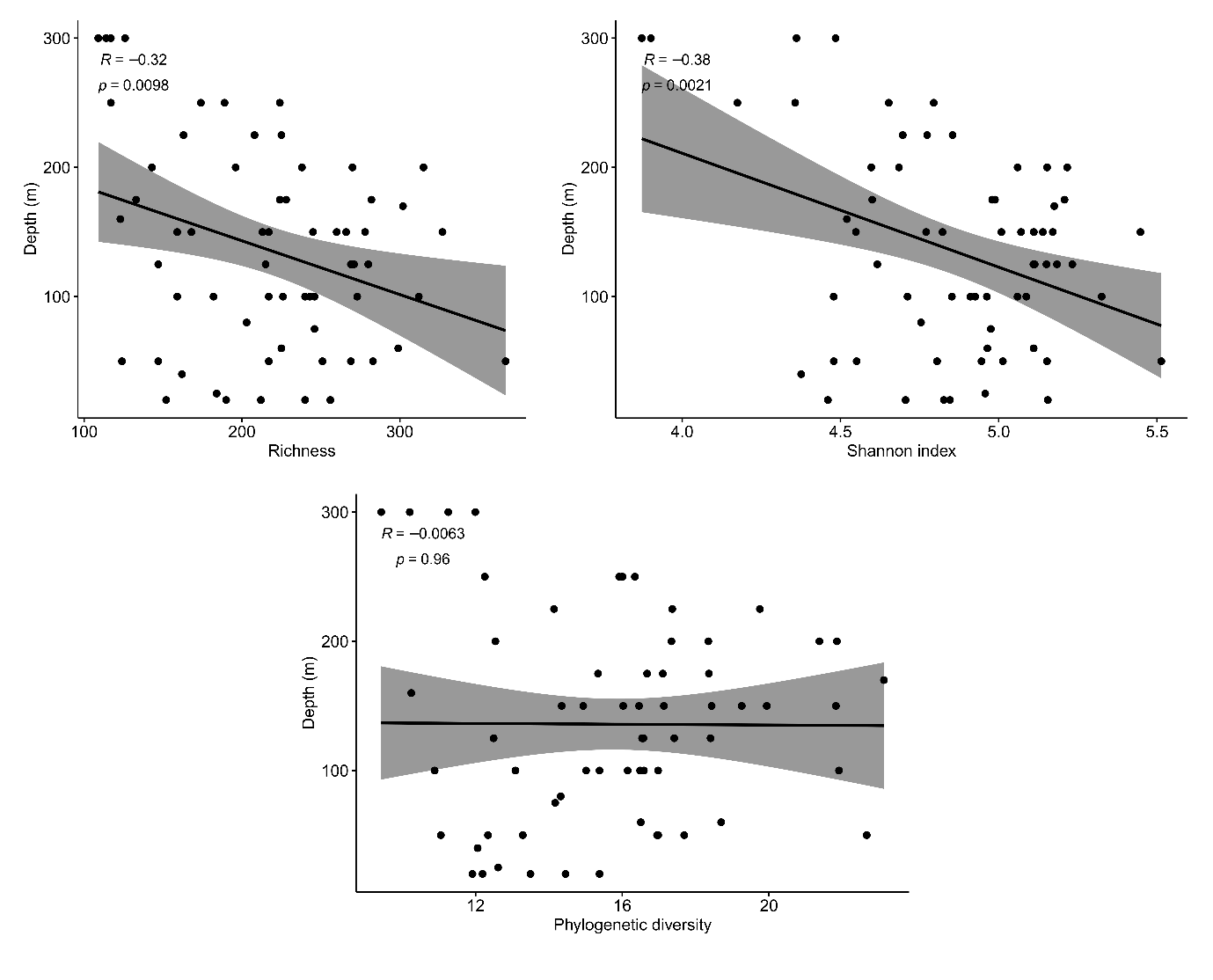


**Fig. S3** The Pearson’s correlation between alpha indices (richness, Shannon index and phylogenetic diversity) of the protistan community and water depth (m).


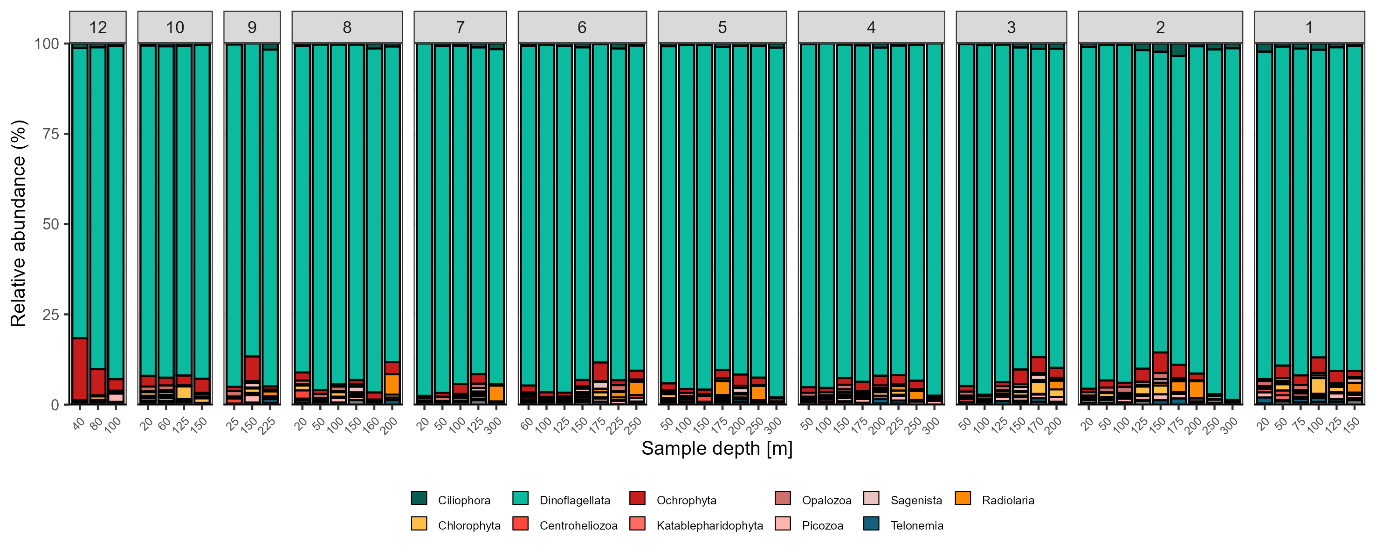


**Fig. S4** Depth distribution of protistan community per sampling station at the division level. The relative sequence abundance of divisions based on 18S rRNA gene amplicon sequencing data within 20 to 300 m.


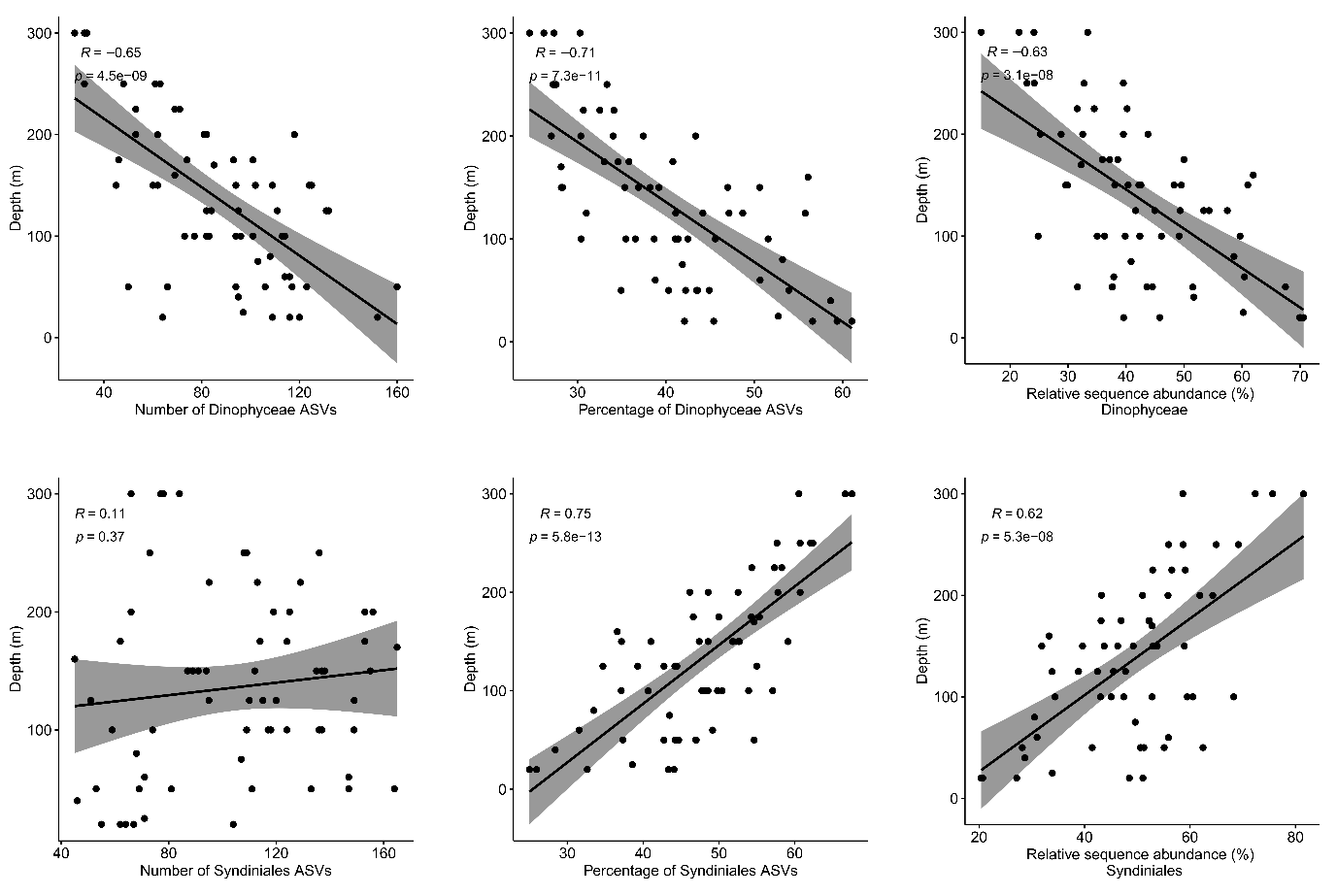


**Fig. S5** The Pearson’s correlation between richness, the relative percentage of ASVs, and relative sequence abundance of Dinophyceae and Syndiniales, and water depth (m).


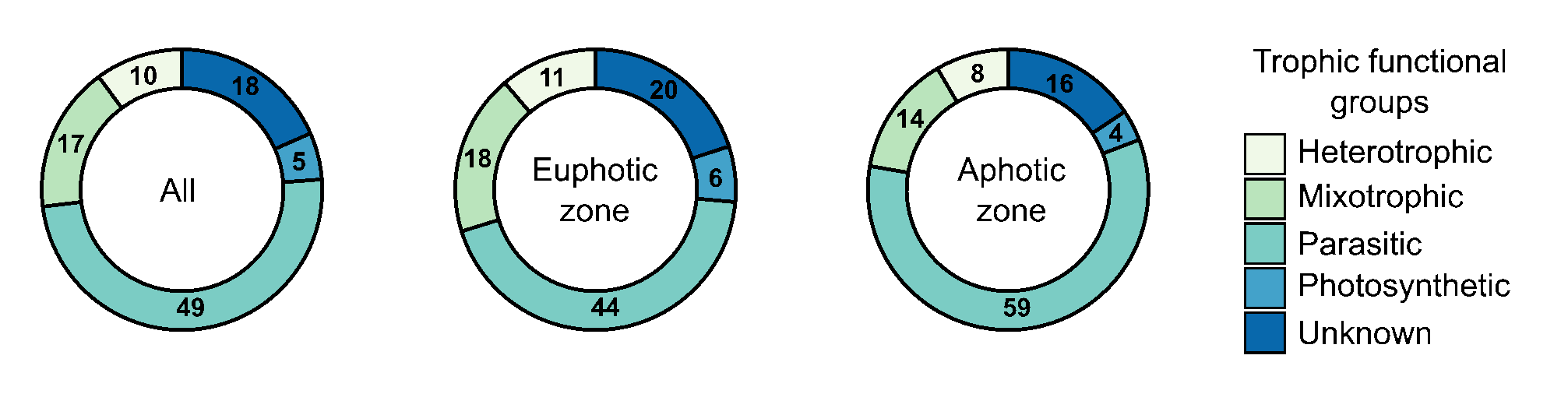


**Fig. S6** Average relative sequence abundance of protistan trophic functional groups in all samples, euphotic and aphotic zone.


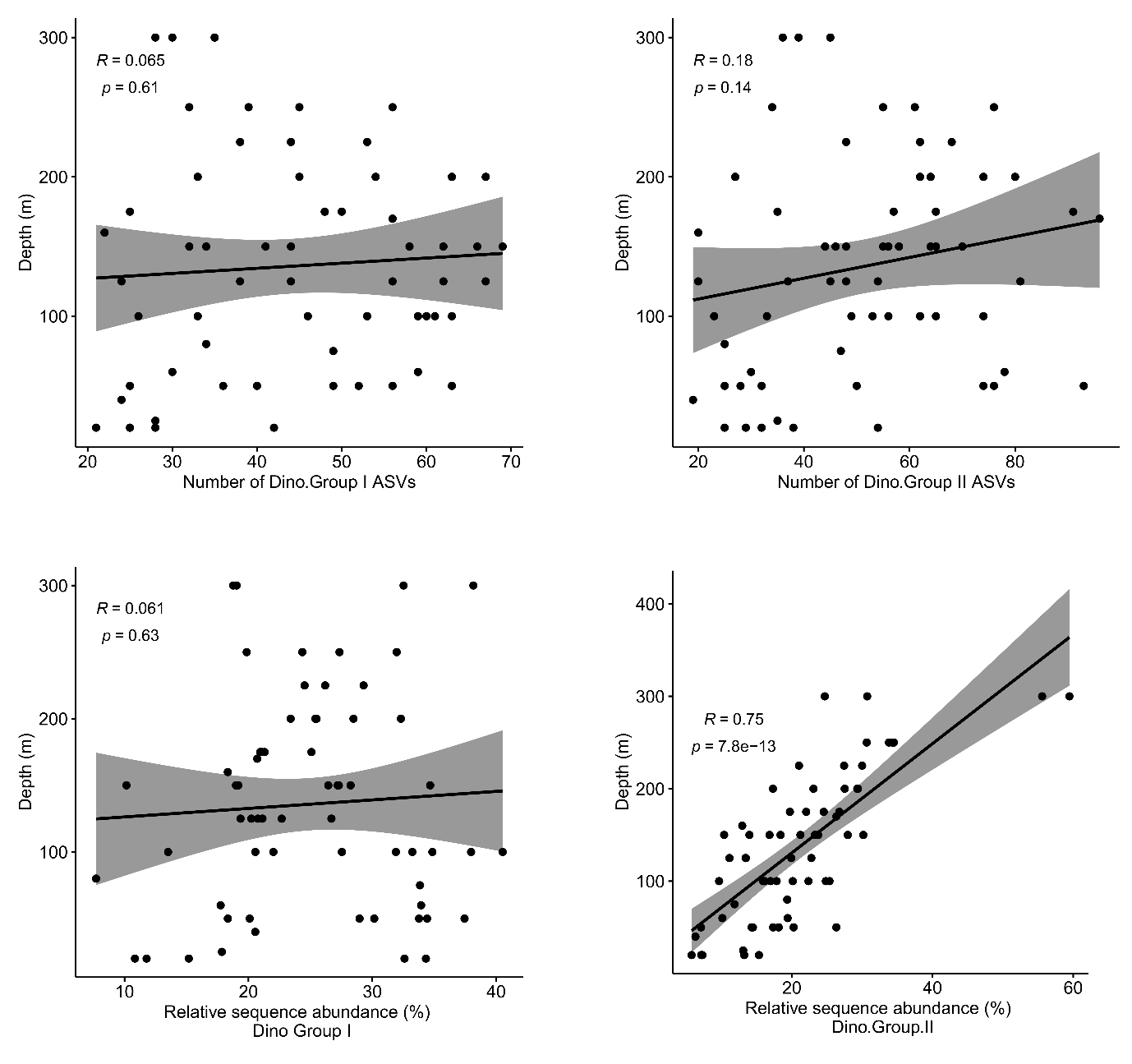


**Fig. S7** The Pearson’s correlation between richness and relative sequence abundance of Dino-Groups I and II, and water depth (m).


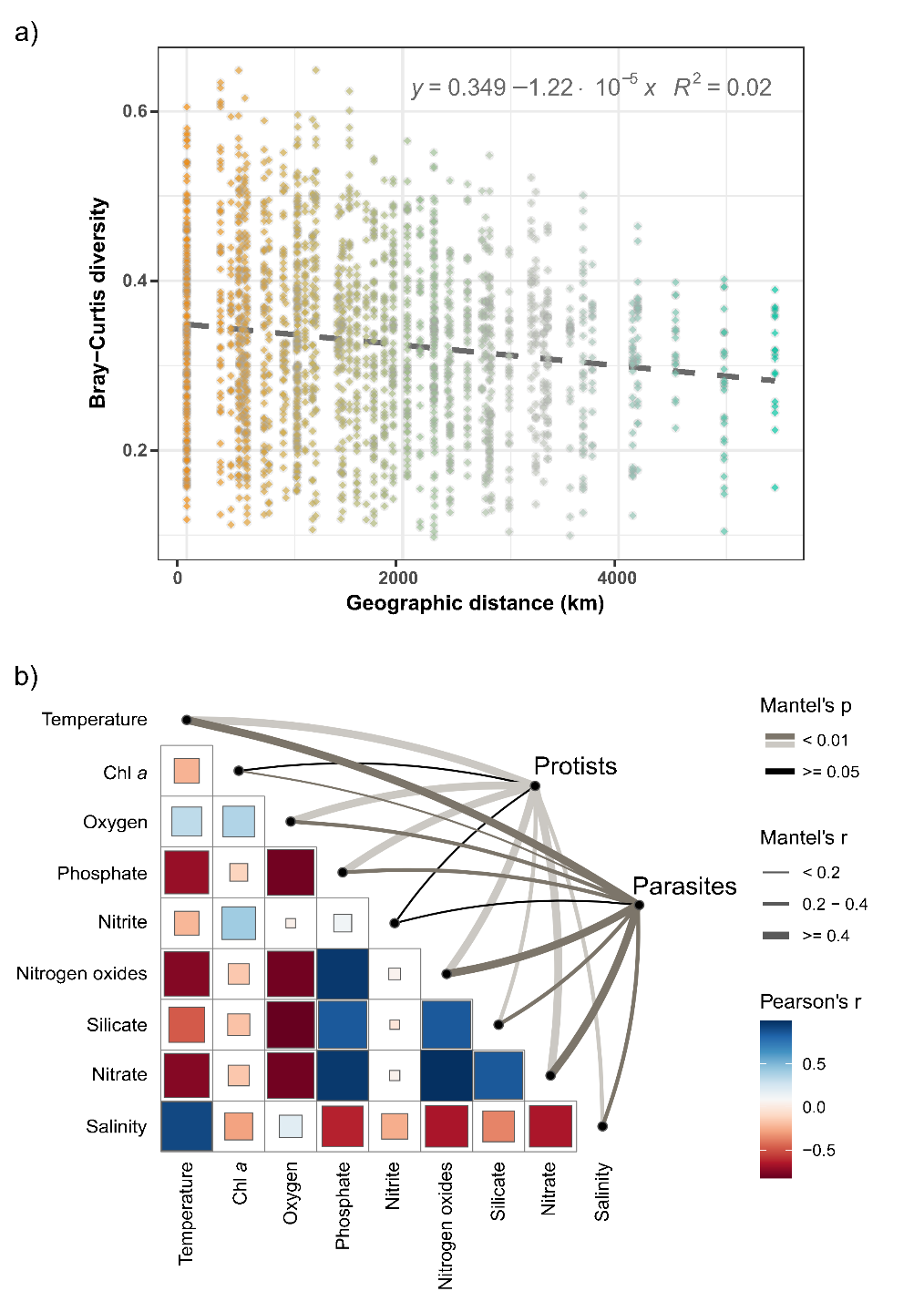


**Fig. S8** Factors driving variations of the protistan community. (A) Distance-decay relationship of Bray-Curtis and geographical distance (km) between sampling stations of the protistan community at the ASV level (p<0.001). The line represents a linear regression. (B) Mantel test correlation plot of protists and parasitic protists with environmental variables based on the Bray-Curtis distance.


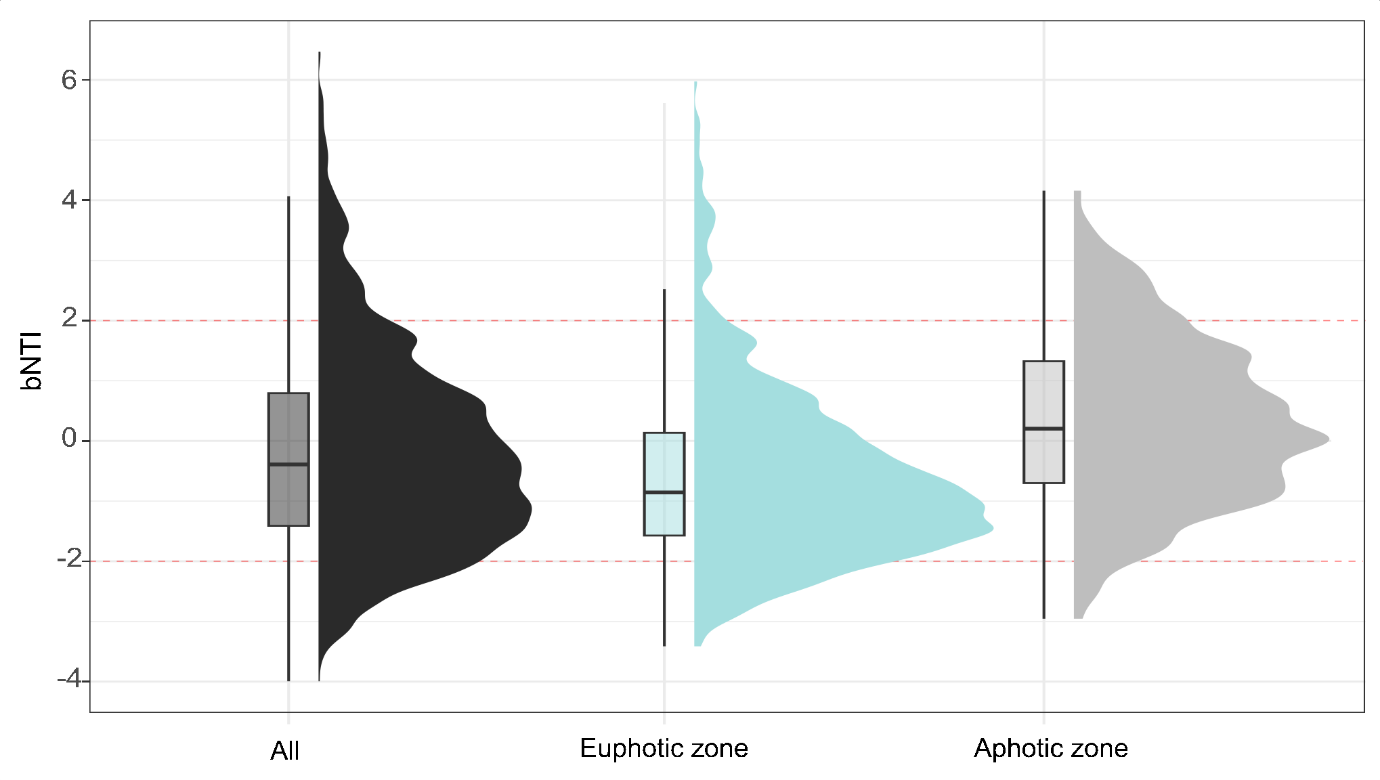


**Fig. S9** βNTI values driving the protistan assembly across the entire, euphotic and aphotic zone. Horizontal dashed red lines indicate upper and lower significance thresholds at βNTI = +2 and -2, respectively.


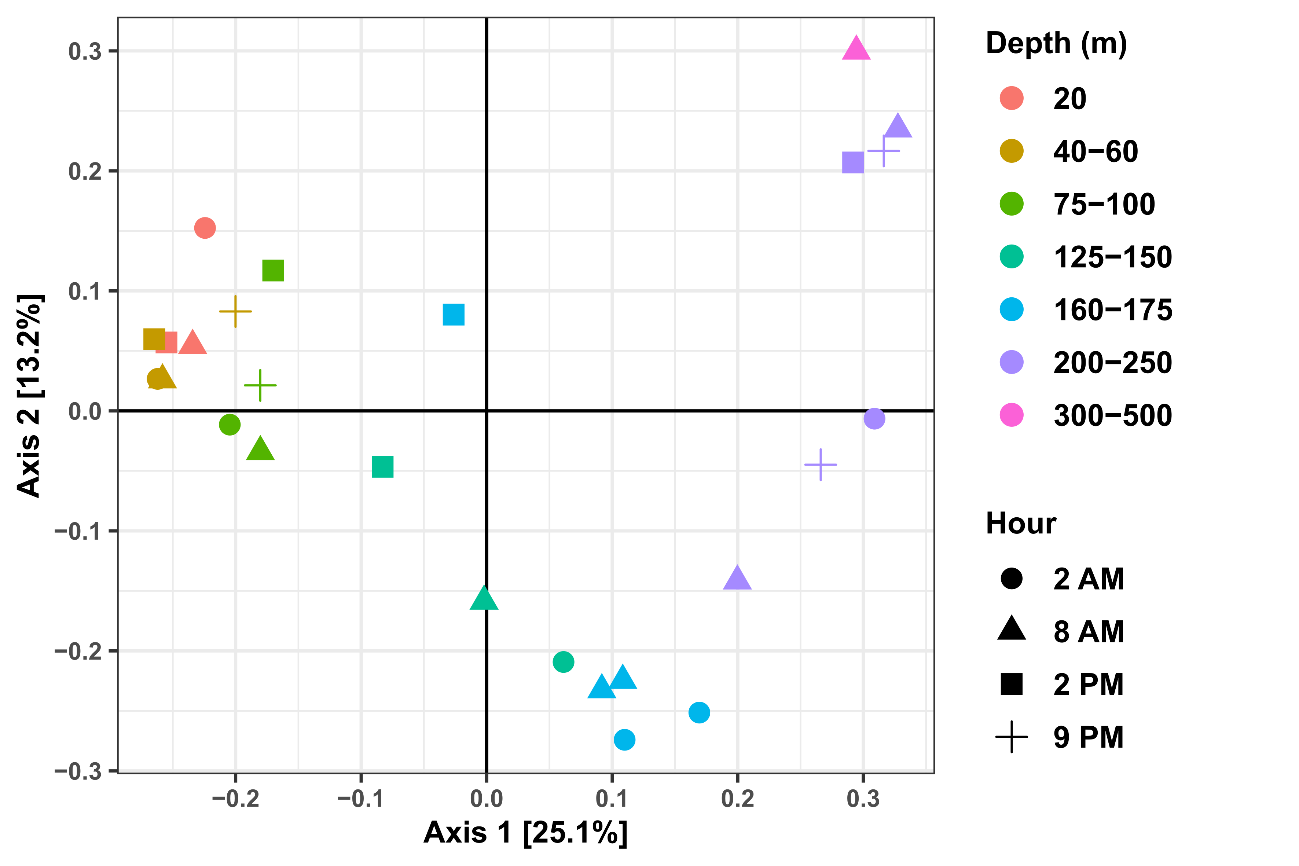


**Fig. S10** PCoA of diel variation of protistan community composition in the central gyre region at station 8 in the vertical profile of 300 m over 24 hours at four time points: 2 AM, 8 AM, 2 PM, and 9 PM. Each point represents an individual sample color-coded by water depth and shape-coded by the sampling hour.


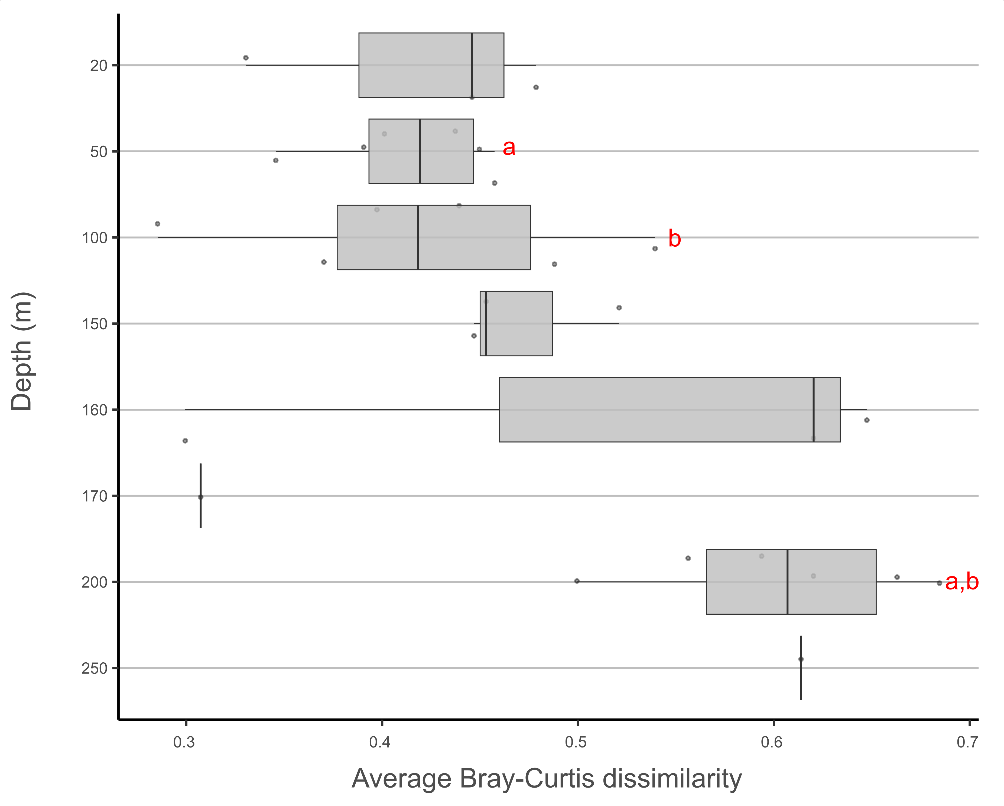


**Fig. S11** Average diel beta diversity per depth based on the Bray-Curtis distance of protistan community composition in the central gyre region at station 8 in the vertical profile of 250 m over 24 hours. Lower-case letters indicate significant differences (p-value < 0.05) among the depths based on one-way ANOVA and a Tukey multiple pairwise comparison test.


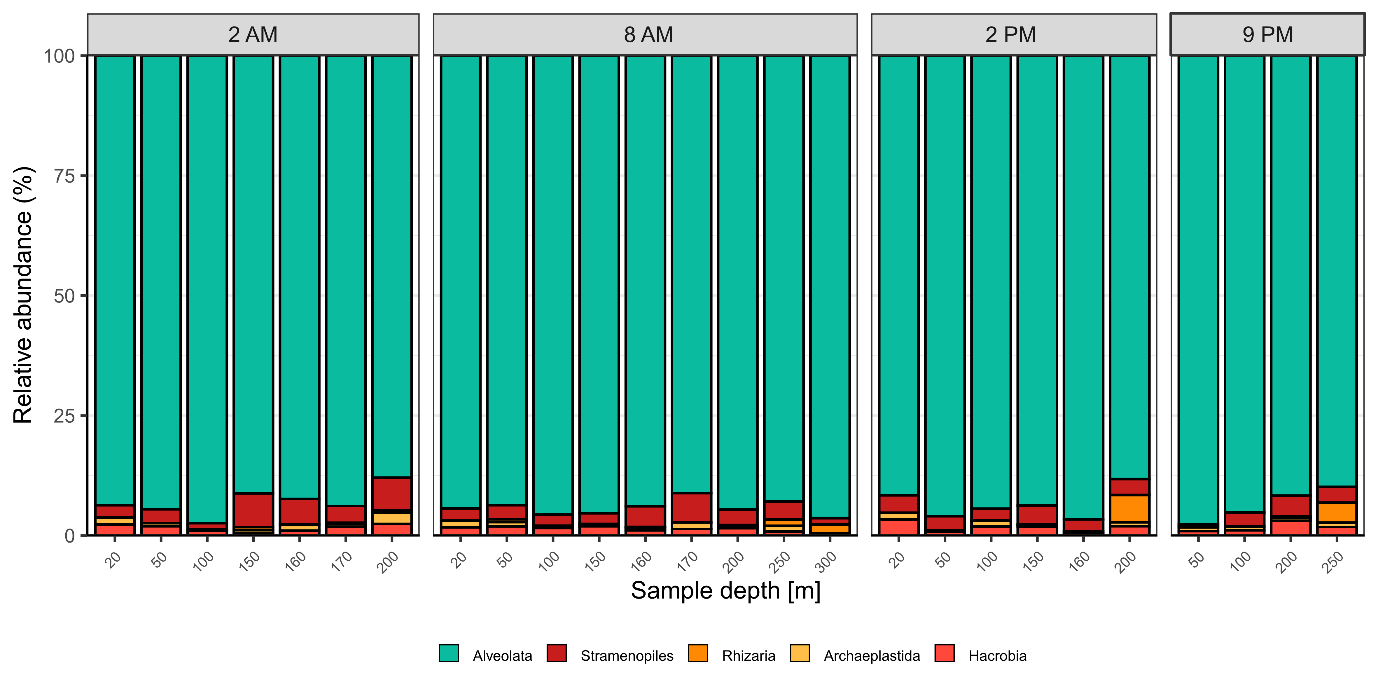


**Fig. S12** Depth distribution of the protistan community at the division level in the central gyre region at station 8 over four time points: 2 AM, 8 AM, 2 PM, and 9 PM. The relative sequence abundance of divisions based on 18S rRNA gene amplicon sequencing data within 20 to 300 m.


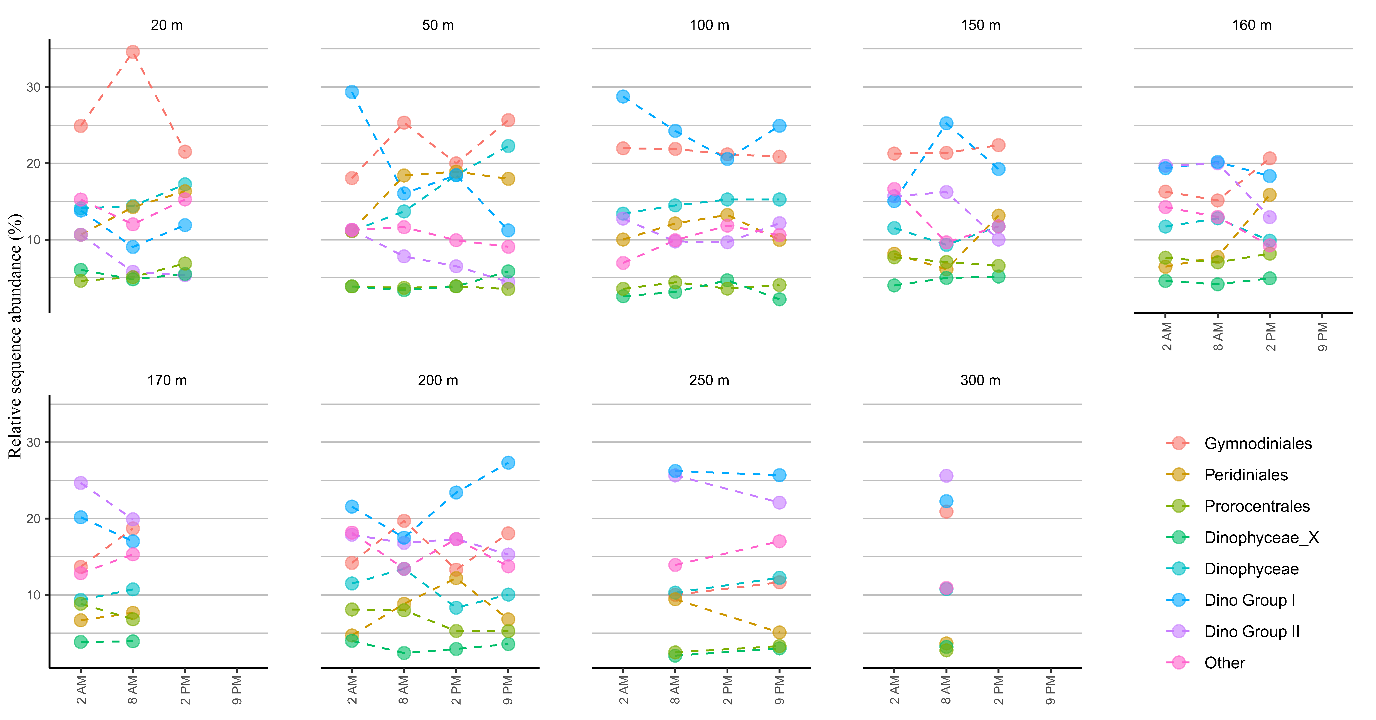


**Fig. S13** Diel variability in relative sequence abundance of protistan community composition at order level based on 18S rRNA gene amplicon sequencing data in the central gyre region at station 8 in the vertical profile of 300 m over 24 hours at four time points: 2 AM, 8 AM, 2 PM, and 9 PM. Orders with relative sequence abundance <1% were aggregated into the group reported as "Other".


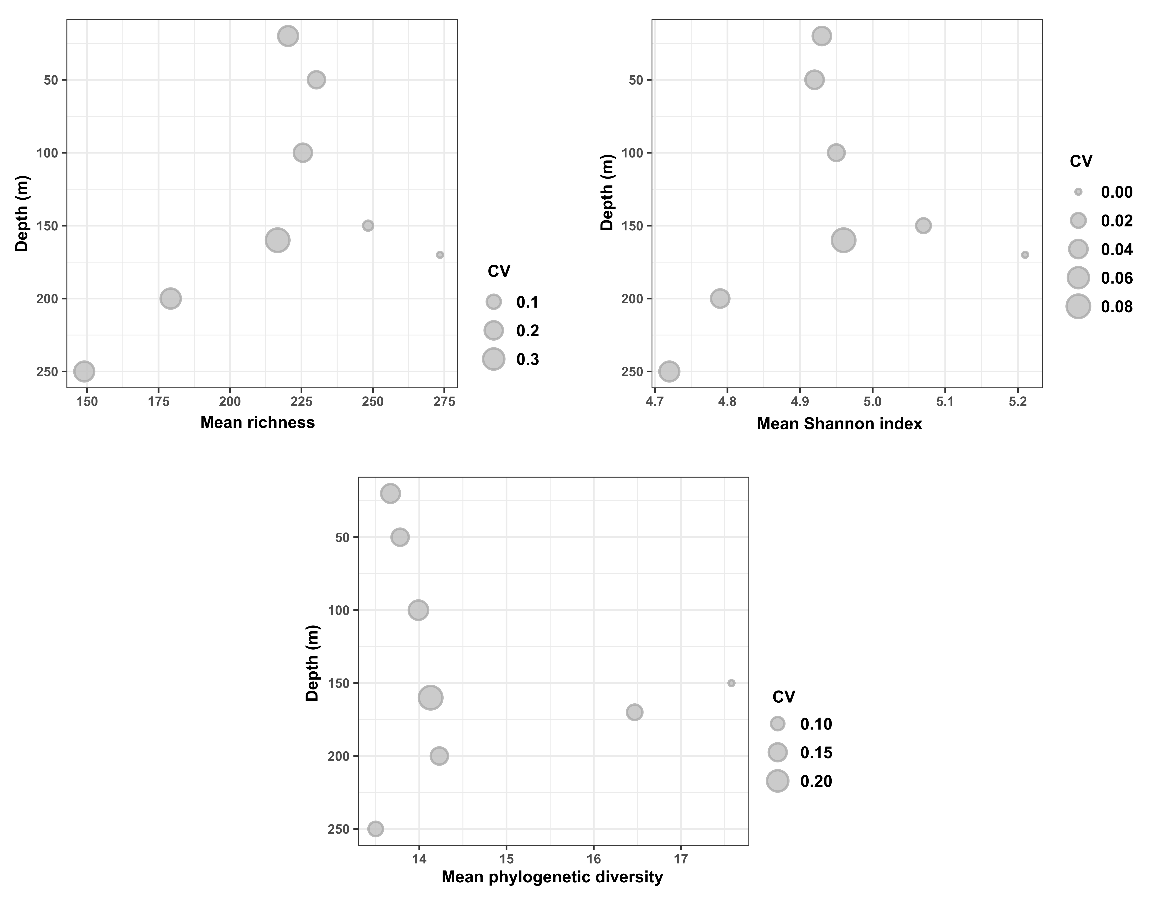


**Fig. S14** Diel vertical variability of alpha indices (richness, Shannon index and phylogenetic diversity) of the protistan community per water depth. Mean alpha diversity values represent the average values of indices per depth. The circle size indicates the variability measured as the coefficient of variation (CV).


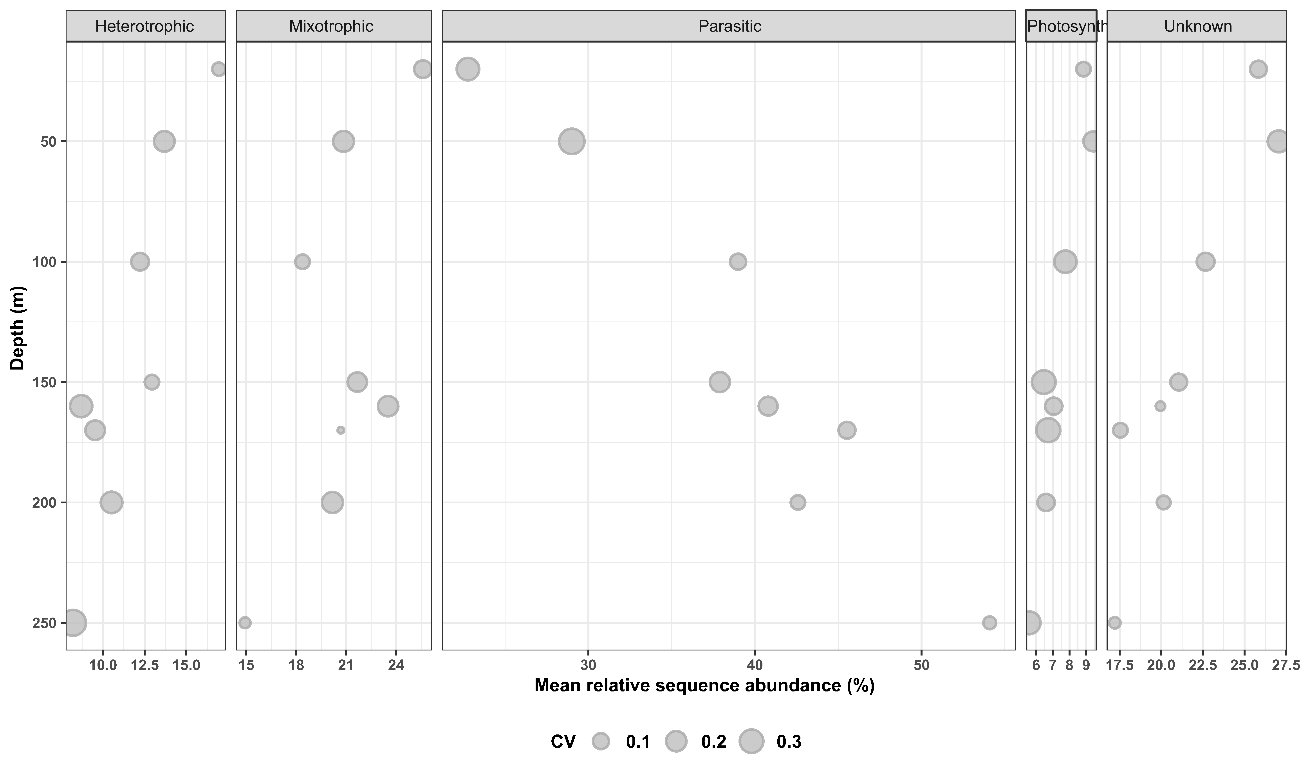


**Fig. S15** Diel vertical variability of protistan trophic functional groups per water depth. Mean relative sequence abundance values represent the average percentages of sequences attributed to each functional group. The circle size indicates the variability measured as the coefficient of variation (CV).


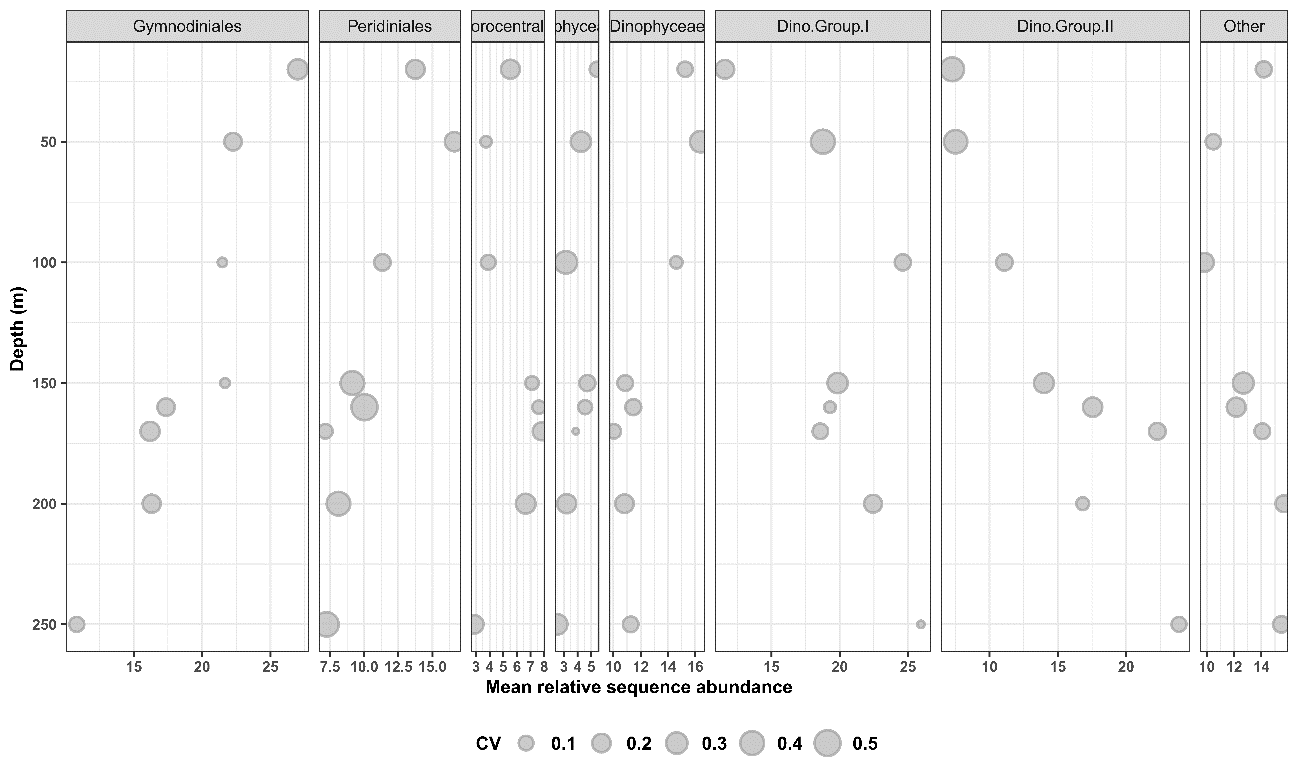


**Fig. S16** Diel vertical variability of protistan community composition at order level per water depth. Mean relative sequence abundance values represent the average percentages of sequences attributed to each order. The circle size indicates the variability measured as the coefficient of variation (CV).

**
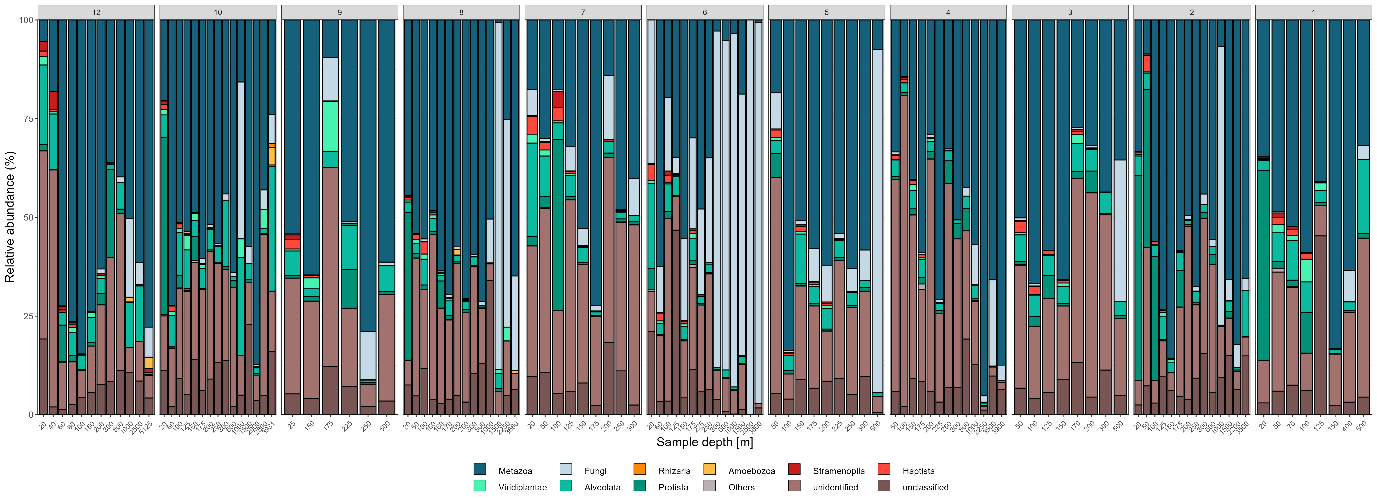
**

**Fig. S17** Relative sequence abundance of kingdoms based on the ITS2 dataset across vertical profile sampling station. Pelagic zones: epipelagic (surface: 20-80 m, DCM: 100-250 m), mesopelagic: 300-500 m, and bathypelagic: 1,000-5,125 m.

**
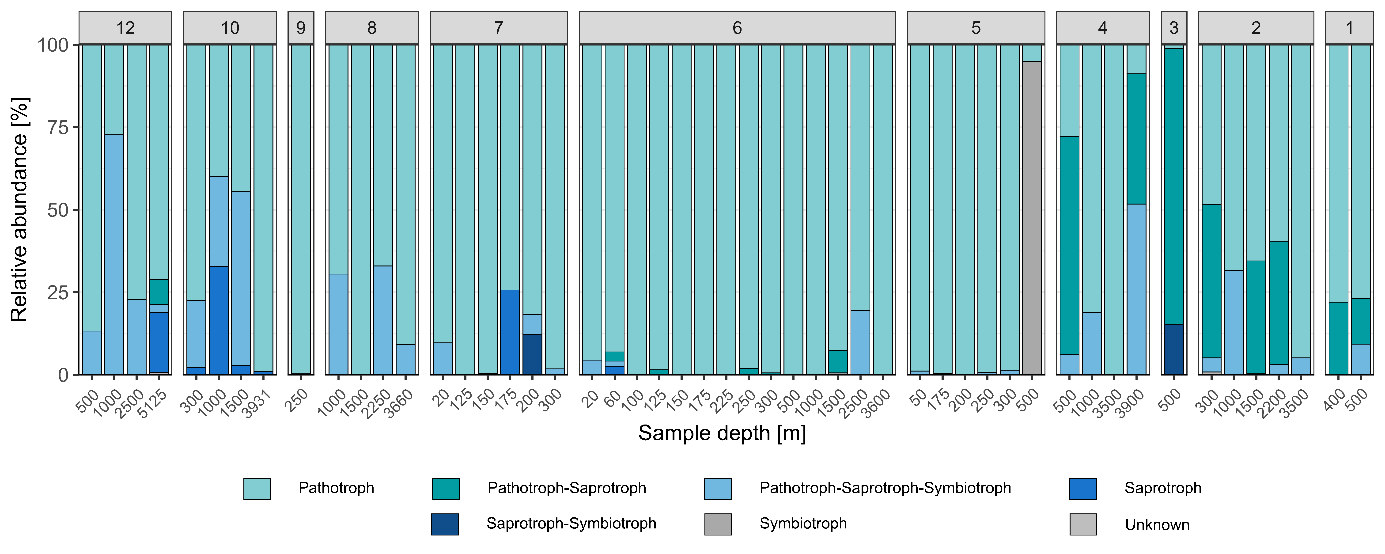
**

**Fig. S18** Relative sequence abundance of fungal trophic functional groups per sampling station and depth. Pelagic zones: epipelagic (surface: 20-80 m, DCM: 100-250 m), mesopelagic: 300-500 m, and bathypelagic: 1,000-5,125 m.
